# Relationship Between Fear of Movement and Physical Activity in Patients With Cardiac, Rheumatologic, Neurologic, Pulmonary, or Pain Conditions: A Systematic Review and Meta-Analysis

**DOI:** 10.1093/ptj/pzaf050

**Published:** 2025-04-06

**Authors:** Miriam Goubran, Ata Farajzadeh, Ian M Lahart, Martin Bilodeau, Matthieu P Boisgontier

**Affiliations:** School of Rehabilitation Sciences, Faculty of Health Sciences, University of Ottawa, Ottawa, Ontario, Canada; Bruyère Health Research Institute, Ottawa, Ontario, Canada; School of Rehabilitation Sciences, Faculty of Health Sciences, University of Ottawa, Ottawa, Ontario, Canada; Bruyère Health Research Institute, Ottawa, Ontario, Canada; Faculty of Education, Health and Wellbeing, Institute of Human Sciences, University of Wolverhampton, Wolverhampton, United Kingdom; School of Rehabilitation Sciences, Faculty of Health Sciences, University of Ottawa, Ottawa, Ontario, Canada; Bruyère Health Research Institute, Ottawa, Ontario, Canada; School of Rehabilitation Sciences, Faculty of Health Sciences, University of Ottawa, Ottawa, Ontario, Canada; Bruyère Health Research Institute, Ottawa, Ontario, Canada; Perley Health Centre of Excellence in Frailty-Informed Care, Ottawa, Ontario, Canada

**Keywords:** Exercise, Health Status, Kinesiophobia, Pain, Prevention, Psychology, Rehabilitation

## Abstract

**Objective:**

Physical activity contributes to the primary, secondary, and tertiary prevention of multiple diseases. However, in some patients, fear of movement may induce avoidance behaviors and reduce engagement in physical activity. This study aims to examine whether this fear of movement is negatively associated with physical activity across several health conditions and what factors may influence this relationship.

**Methods:**

Five databases were searched for studies including both a measure of fear of movement and physical activity. Two reviewers screened articles for inclusion, assessed risk of bias, and extracted data from each study. Pearson product-moment correlations were pooled from eligible studies using the generic inverse pooling and random effects method to examine the relationship between fear of movement and physical activity.

**Results:**

Seventy-four studies were included in the systematic review and 63 studies (83 estimates, 12,278 participants) in the main meta-analysis. Results showed a negative correlation between fear of movement and physical activity (*r* = −0.19 [95% CI = −0.26 to −0.13]; *I*^2^ = 85.5%). Funnel plot analysis showed evidence of publication bias, but *p*-curve analysis suggested that our results could not be caused by selective reporting. A subgroup meta-analysis showed that the correlation was statistically significant in patients with cardiac, rheumatologic, neurologic, or pulmonary conditions but not in patients with chronic or acute pain.

**Conclusions:**

Our results suggest that higher levels of fear of movement are associated with lower levels of physical activity in several health conditions that are not necessarily painful.

**Impact:**

Fear of movement should be dissociated from pain and considered in relation to specific health conditions when implementing exercise therapy. Fear of movement may have prognostic and therapeutic implications in patients for whom physical activity contributes to prevent recurrence or worsening of their condition.

## INTRODUCTION

Seven decades ago, the seminal work of Morris et al^1^ showed that conductors on London double-decker buses, who were responsible for checking tickets, assisting passengers with luggage and supervising the loading and unloading of passengers, had a lower incidence and less severe coronary heart disease than bus drivers. Since then, the scientific literature demonstrating the health benefits of physical activity has grown exponentially and expanded to include multiple health conditions.^2^ These benefits include reduced risk of disability, disease, and mortality.^2^^,^^3^ Specifically, higher levels of physical activity contribute to reducing the risk of cardiovascular disease,^4^ obesity,^5^ depression,^6^ hypertension,^7^ cancer,^8^ and dementia.^9^ Yet, 1 in 4 adults worldwide does not meet the recommendations for physical activity.^10^ Physical activity also plays an important role in secondary and tertiary prevention by reducing the impact, slowing the progression, and preventing the recurrence of multiple health conditions, including cardiovascular disease,^11^^,^^12^ osteoarthritis,^13^ stroke,^14^^,^^15^ and cancer.^16^

Several factors may explain physical inactivity,^17^ including environmental, interpersonal, and intrapersonal factors.^18^ Environmental factors include lack of access, weather conditions, and safety concerns.^19^ Interpersonal factors include family responsibilities, lack of support, and lack of a gym partner.^20^ Intrapersonal factors include gender,^21^ age,^22^ cognitive function,^23^^,^^24^ and socioeconomic circumstances.^25^ Another intrapersonal factor of interest is fear of movement, which can be defined as an excessive, irrational, and debilitating fear of movement and activity resulting from a sense of vulnerability to pain, injury, or a medical condition.^26^ Fear of movement is typically measured using self-administered questionnaires, such as the Tampa Scale for Kinesiophobia (TSK),^27^^,^^28^ which assesses an individual’s belief that physical activity can lead to injury or pain and that the severity of their medical condition is underestimated. Although fear of movement is often observed in the context of pain or a clinical condition, its presence in adults who are otherwise healthy is also possible^29^ because of the irrational nature of this condition. The irrational fear that characterizes fear of movement is likely to influence the desires and impulses for movement and rest,^30^ as well as affective determinants of physical activity in general.^31^ Here, we consider that fear of movement may result not only from the accumulation of overwhelming emotions over time that develop into a phobia (ie, kinesiophobia) but also from automatic processes, such as conditioning, or from learning processes based on the integration of perceptual and environmental information.

The relationship between fear of movement and physical activity can be explained by theories suggesting that the perception of a cue related to physical activity automatically activates the concept of physical activity as well as the unpleasant (or pleasant) affective memories associated with this concept.^32–35^ This activation results in an impulse that favors the tendency to avoid (or approach) physical activity.^36^ Thus, negative affective associations are likely to hinder physical activity. Accordingly, an aversive fear of pain, injury, or aggravation of a medical condition that has been associated with the concept of movement may result in the development of automatic avoidance behaviors that contribute to the maintenance and exacerbation of this fear and ultimately lead to a diminished ability to engage in regular physical activity.

Previous systematic syntheses of the literature on this topic include a meta-analysis^37^ and 2 systematic reviews.^38^^,^^39^ The main results of these reviews suggest that exercise interventions may reduce fear of movement in individuals with back pain. Although back pain is one condition that may contribute to fear of movement, it is not the only one. The relationship between physical activity and fear of movement should be investigated in other conditions, such as cardiac, neurologic, and rheumatologic conditions.

The main objective of this study was to systematically review the literature and conduct a meta-analysis of the direct relationship between fear of movement and physical activity. We hypothesized that levels of fear of movement would be negatively associated with levels of physical activity. In addition, we examined the moderating effect of health status, physical activity measurement instruments (ie, accelerometers, pedometers, questionnaires), physical activity outcomes (eg, total physical activity, moderate or vigorous physical activity, steps per day), and fear of movement measurement instruments. Finally, because fear of movement and physical activity can vary with age, sex, and pain,^40^^,^^41^ we explored the influence of these factors on the association between fear of movement and physical activity.

## METHODS

### Search Strategy

This review was reported according to the Preferred Reporting Items for Systematic Reviews and Meta-Analyses (PRISMA) guidelines.^42^ Potential studies were identified by searching the MEDLINE (via PubMed), PsycINFO, CINAHL, EMBASE, and SPORTDiscus databases. In October 2023, 2 reviewers (M.G. and A.F.) searched for all available records using the following combination of keywords in the title or abstract of the article: (“kinesiophobia” OR “fear avoidance” OR “fear of movement” OR “movement phobia” OR “movement fear”) AND (“physical activity” OR “exercise” OR “walking”). In PsycINFO, the limits “clinical trial,” “quantitative study,” “peer-reviewed journal,” “English,” and “human” were used. In PubMed the limits “clinical trial,” “observational study,” “RCT,” and “English” were used. In SPORTDiscus the limits “peer-reviewed,” “English,” “academic journal,” and “article” were used. In CINAHL, the limits “peer-reviewed,” “English,” “research article,” “journal article,” and “humans” were used. To reduce literature bias,^43^^,^^44^ this systematic review was preregistered at the International Prospective Register of Systematic Reviews (PROSPERO).^45^

### Eligibility Criteria and Study Selection

#### Inclusion Criteria

To be included in this systematic review, articles had to be published in a peer-reviewed journal, be written in English, report original data collected from human participants, include at least 1 self-reported measure of fear of movement and 1 measure of physical activity, and formally test the association between these 2 variables, be it a univariate or multivariate test. The physical activity measure could be derived from a self-reported measure of the level of physical activity or from a device (eg, accelerometer, pedometer) worn while participants are engaged in their normal daily activities.

#### Exclusion Criteria

Studies were excluded if they were published as a book chapter, study protocol, or conference abstract or were based on laboratory-based measures of physical fitness (eg, maximal muscle force, maximum oxygen consumption) and not on a measure of physical activity.

#### Study Selection

Article screening was performed in Covidence systematic review software (Veritas Health Innovation, Melbourne, Victoria, Australia; www.covidence.org), a web-based collaborative software platform that streamlines the production of systematic reviews. After removing duplicates, titles and abstracts were independently reviewed by 2 reviewers (M.G., A.F.) according to the inclusion and exclusion criteria using a systematic 5-step process. If there was any doubt at any step, the full text was further reviewed. In step 1, articles not written in English were excluded. In step 2, articles that did not report original empirical data were excluded (eg, reviews, meta-analyses, commentaries, technical reports, case studies). In step 3, articles that did not involve human participants were excluded. In step 4, articles that did not assess both fear of movement and physical activity were excluded. In step 5, articles that did not formally test the association between fear of movement and physical activity were excluded. In addition, we performed reference screening and forward citation tracking on the articles remaining after step 5. Any disagreements between the 2 reviewers were resolved by consensus among 3 reviewers (M.G., A.F., M.P.B.).

### Data Extraction

Data extracted from selected articles included first author’s name, article title, publication year, digital object identifier, number of participants, number of men and women, age range, mean age, mean weight, mean height, mean body mass index, health status, mean pain intensity, type of fear of movement measure, level of fear of movement, type of physical activity measure, type of physical activity outcome, and level of physical activity (continuous or categorical), as well as statistical estimates and significance of the association between fear of movement and physical activity.

### Methodological Quality and Risk-of-Bias Assessment

The risk of bias of the studies included in the systematic review was estimated using the National Institutes of Health Quality Assessment Tool for Observational Cohort and Cross-Sectional Studies,^46^ the Transparent Reporting of Evaluations with Non-Randomized Designs (TREND) reporting checklist,^47^ and the Consolidated Standards of Reporting Trials (CONSORT) reporting checklist for randomized trials.^48^ All scores were normalized to a scale from 0 to 10 to make them comparable across assessment instruments (Suppl. Table 1), with higher scores reflecting a lower risk of bias and better study quality.

### Meta-Analysis

All analyses were performed in the R Studio integrated development environment (2023.06.1 + 524; “Mountain Hydrangea” release) for the R software environment^49^ using the meta^50^ and metafor^51^^,^^52^ R packages.^53^

#### Main Meta-Analysis

We pooled Pearson product-moment correlations from eligible studies to examine the relationship between fear of movement and physical activity. Correlations were pooled using the generic inverse pooling method via the metacor function in the meta R package.^50^ This function automatically performs a necessary Fisher *z* transformation on the original, untransformed correlations prior to pooling. The metacor function also reconverts the pooled association back to its original form for ease of interpretation. Correlation estimates were nested within studies using the cluster argument to account for the dependencies between these estimates, resulting in a 3-level meta-analysis (level 1: participants; level 2: correlation estimates; level 3: studies). The distribution of variance across levels was assessed using the multilevel version of *I^2^*.^54^ The performance of the 2-level and 3-level meta-analyses was assessed and compared using the metafor R package.^51^^,^^52^

We anticipated considerable between-study heterogeneity and therefore used a random-effects model to pool correlations. The restricted maximum-likelihood estimator^55^ was used to calculate the heterogeneity variance τ-squared. In addition to τ-squared, to quantify between-study heterogeneity, we report the *I*^2^ statistic, which provides the percentage of variability in the correlations that is not caused by sampling error.^56^ The *I*^2^ statistic was interpreted as follows: 0% to 40%, may not be important; 30% to 60%, may represent moderate heterogeneity; 50% to 90%, may represent substantial heterogeneity; and 75% to 100%, may represent considerable heterogeneity. To reduce the risk of false-positive results, we used a Knapp-Hartung adjustment^57^ to calculate the confidence interval around the pooled association. We also report the prediction interval, which provides a range within which we can expect the associations of future studies to fall based on the current evidence.^58^

#### Publication Bias Assessment

Publication bias was assessed using a funnel plot, which is a scatterplot of the studies’ effect size expressed as the Fisher *z*-transformed correlation on the *x*-axis against a measure of their standard error (SE; which is indicative of precision of the study’s effect size) on the *y*-axis. When there is no publication bias, the data points in a funnel plot should form a roughly symmetrical, upside-down funnel. Studies in the top part of the plot, which have lower SEs, are expected to lie closely together and not far away from the pooled effect size. In the lower part of the plot, studies have higher SEs, the funnel “opens up,” and effect sizes are expected to scatter more heavily to the left and right of the pooled effect. Egger regression^59^ can be used to formally test the asymmetry of the funnel plot. However, since there is no direct function to conduct the Egger test for 3-level models, we calculated it by using the SEs of the effect size estimates as a predictor in the meta-regression.^60^

*p*-curve analysis^61^ was conducted to assess whether the distribution of the statistically significant results was consistent with what would be expected if only true effects were present. When the null hypothesis is true (ie, there is no true effect), *P* values are assumed to follow a uniform distribution: Highly significant effects (eg, *P* = .01) are as likely as barely significant effects (eg, *P* = .049). However, when the null hypothesis is false (ie, there is a true effect in our data), *P* values are assumed to follow a right-skewed distribution: highly significant effects are more likely than barely significant effects. A left-skewed distribution would suggest that some studies used statistical tests to find significant results in ways that may not be reproducible or generalizable (ie, *p*-hacking).

#### Secondary Meta-Analysis

A secondary meta-analysis was conducted using the same approach, but based on Spearman ρ values, to further test the relationship between fear of movement and physical activity.

#### Subgroup Analyses and Meta-Regressions

Subgroup analyses were conducted to examine the differences in correlations between studies including participants with different health conditions and using different types of physical activity measures (ie, device-based vs self-reported), physical activity measurement instruments (ie, type of questionnaires, type of devices), physical activity outcomes, and fear of movement measures.

Exploratory meta-regressions were conducted to examine whether the average age of participants, the proportion of women, and the level of pain in a study predicted the reported correlation between fear of movement and physical activity. Pain was normalized to a scale from 0 to 100 to make the data comparable across pain scales. A sensitivity analysis was conducted to examine whether the quality of the studies affected the results.

### Role of the Funding Source

The funders had no role in the data collection, management, analysis and interpretation, writing of the report, or the decision to submit the report for publication.

## RESULTS

### Literature Search

**Figure 1:**
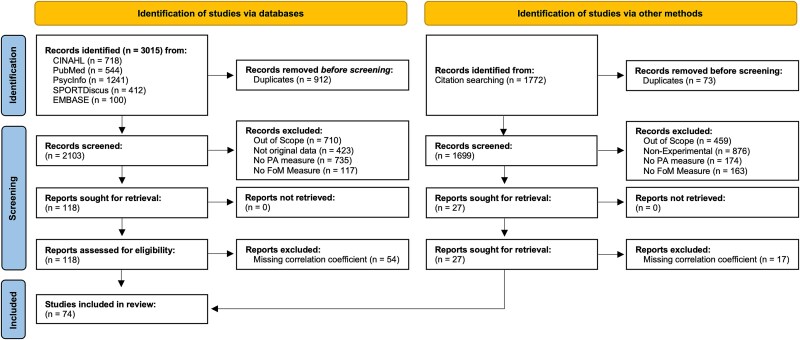
Preferred Reporting Items for Systematic Reviews and Meta-Analyses (PRISMA) 2020 Flow Diagram. Abbreviations: FoM = Fear of Movement; PA = Physical Activity.

The primary search identified 3015 potentially relevant articles from the 5 databases (Figure 1), including 912 duplicates. Of the 2103 articles screened, disagreement occurred in 210 cases (10%), all of which were resolved by consensus. All articles remained after step 1 as they were all written in English. A total of 1133 articles were excluded in step 2 because they were irrelevant (*n =* 710) or did not report original data (*n =* 423). No articles were excluded in step 3 because they all involved human participants. Eight hundred fifty-two articles were excluded in step 4 because they did not assess fear of movement (*n =* 117) or physical activity (*n =* 735). Seventy-seven articles were initially excluded at step 5 because they did not formally test the correlation between fear of movement and physical activity or did not report the estimate of this correlation. However, the corresponding authors of these articles were contacted by email to request the Pearson correlation estimate of this association and the sample size used to calculate it. Nineteen authors replied to our email: Eight authors provided raw data for 10 studies^62–71^ and 11 authors provided the Pearson correlation estimate.^29^^,^^72–81^ In addition, the Pearson correlation estimate of 2 articles was calculated based on the information reported in the article.^82^^,^^83^ This process reduced the number of studies excluded at step 5 to 54, resulting in a total of 64 articles included from the databases.

Using reference screening and forward citation tracking, the authors identified 27 studies that assessed both physical activity and fear of movement, of which 8 reported an estimate of their relationship^84–91^ and 19 did not.^92–110^ The corresponding authors of these 19 studies were asked by email to provide this estimate or their data. Two authors sent the estimate,^104^^,^^107^ but the remaining 17 emails were left unanswered.^92–103^^,^^105^^,^^106^^,^^108–110^

### Descriptive Results

#### Participants

The 74 articles identified by the systematic review included a total of 13,388 participants who were 11 to 85 years old, including 7308 women, 4729 men, and 1351 participants whose gender and sex were not reported. The studies examined populations with pain (*n =* 37)^62^^,^^64–71^^,^^73^^,^^76–81^^,^^83^^,^^88^^,^^89^^,^^111–128^ cardiac conditions (*n =* 6),^86^^,^^87^^,^^104^^,^^129–131^ surgery (*n =* 8),^63^^,^^131–137^ arthritis (*n =* 10),^75^^,^^82^^,^^84^^,^^85^^,^^91^^,^^138–142^ neurologic conditions (*n =* 3),^90^^,^^143^^,^^144^ pulmonary conditions (*n =* 3),^145–147^ cancer (*n =* 1),^74^ and women’s health conditions (*n =* 2),^72^^,^^148^ as well as adults who were healthy (*n =* 6)^29^^,^^85^^,^^107^^,^^149–151^ (Suppl. Table 1).

#### Fear of Movement

In 54 of the 74 studies, fear of movement was assessed using the 17-item TSK (TSK-17 [*n =* 38^29^^,^^62^^,^^64^^,^^65^^,^^68^^,^^72^^,^^73^^,^^77^^,^^84^^,^^85^^,^^90^^,^^91^^,^^111–114^^,^^117–120^^,^^122–125^^,^^130^^,^^133–135^^,^^138–144^^,^^146^^,^^148^^,^^150^]), shorter versions of the TSK (11-item TSK [TSK-11]^152^ [*n =* 10^63^^,^^81^^,^^88^^,^^116^^,^^121^^,^^122^^,^^128^^,^^131^^,^^136^^,^^151^], 14-item TSK [TSK-14] [*n =* 1^74^], 7-item TSK [TSK-7] [*n =* 1^145^], 13-item TSK [TSK-13]^153^ [*n =* 2^115^^,^^137^]), or an adaptation for patients with coronary artery disease (TSK-Heart^154^ [*n =* 2^86^^,^^87^]). The TSK is a questionnaire that assesses the belief that movement can lead to (re) injury, pain, or aggravation of an underlying and serious medical condition.^28^ Each item is rated on a Likert scale ranging from 1 (strongly disagree) to 4 (strongly agree). On the TSK-17, a score of 37 is used to distinguish between low (≤37) and high (>37) levels of fear of movement.^27^ On the TSK-13, scores inferior to 23 are considered subclinical.^155^ The other measures that were used are the Fear Avoidance Beliefs Questionnaire^156^ (*n =* 15),^66^^,^^67^^,^^69–71^^,^^76^^,^^78–80^^,^^82^^,^^83^^,^^104^^,^^126^^,^^127^^,^^132^ the Kinesiophobia Causes Scale^157^ (*n =* 2),^107^^,^^149^ the Fear of Activities in Situations Scale (*n =* 1),^129^ the Brief Fear of Movement Scale for Osteoarthritis^158^ (*n =* 1),^75^ and the Breathlessness Beliefs Questionnaire (*n =* 1).^147^

Sixty-four studies reported mean levels of fear of movement (Suppl. Table 1). The studies based on the TSK-17 or TSK-Heart (mean range: 17-68) reporting the highest levels of fear of movement were those involving participants with a cardiovascular condition (41.4-49.7), followed by studies testing participants with arthritis (31.8-45.27) or chronic pain (30.5-44.6). Levels of fear of movement were lower in participants with a neurologic (36.6-41), pulmonary (20.7-39.6), women’s health (36), or surgical (32.9-35.9) condition and in adults who were healthy (18.9-39.0).

#### Physical Activity

Fifty-one studies assessed physical activity using a self-reported measure (Suppl. Table 1). Most of these questionnaire-based studies used the short form of the International Physical Activity Questionnaire (*n =* 20),^29^^,^^73^^,^^76^^,^^78^^,^^79^^,^^82^^,^^85^^,^^86^^,^^112^^,^^114^^,^^116^^,^^130^^,^^131^^,^^133^^,^^138–141^^,^^146^^,^^147^ which consists of 6 items assessing time spent in light (eg, walking), moderate (eg, carrying light loads, cycling at moderate speed, doubles tennis), and vigorous (eg, digging, fast cycling, heavy lifting, aerobics) physical activity over the last 7 days.^159^ Other questionnaires used to assess physical activity were the Baecke Habitual Physical Activity Questionnaire^160^ (*n =* 5),^64^^,^^89^^,^^90^^,^^107^^,^^113^ the Saltin-Grimby Physical Activity Level Scale^161^ (*n =* 5),^80^^,^^104^^,^^115^^,^^125^^,^^135^ the Godin-Shephard Leisure-Time Exercise Questionnaire^162^ (*n =* 2),^121^^,^^134^ the Minnesota Leisure Time Physical Activity Questionnaire^163^ (*n =* 1),^136^ the Physical Activity Scale for the Elderly^164^ (*n =* 2),^123^^,^^149^ the Physical Activity Questionnaire for the Elderly^164^ (*n =* 1),^72^ the Short Questionnaire to Assess Health Enhancing Physical Activity^165^ (*n =* 1),^142^ the Tegner Assessment Scale^166^ (*n =* 1),^63^ the University of California–Los Angeles activity score^167^ (*n =* 1),^84^ the Leisure Time Physical Activity Index^168^ (*n =* 1),^67^ the Global Physical Activity Questionnaire^169^ (*n =* 2),^77^^,^^83^ the Freiburger Questionnaire on Physical Activity^170^ (*n =* 1),^127^ the Jurka Physical Activity Scale^171^ (*n =* 1),^81^ the Rapid Assessment of Physical Activity Questionnaire^172^ (*n =* 1),^68^ the Tecumseh Occupational Activity Questionnaire^173^ (*n =* 1),^136^ and the Australian Health Survey–derived questions (*n =* 1).^71^

Physical activity was also assessed with devices such as accelerometers measuring accelerations in 3 dimensions (*n =* 23)^65^^,^^66^^,^^74^^,^^75^^,^^78^^,^^91^^,^^110^^,^^113^^,^^117^^,^^118^^,^^120^^,^^122^^,^^124^^,^^126^^,^^129^^,^^132^^,^^137^^,^^143–145^^,^^148^^,^^150^^,^^151^ and pedometers measuring the number of steps (*n =* 3)^63^^,^^88^^,^^128^ (Suppl. Table 1). In most studies, the device was worn at the hip (*n =* 10).^63^^,^^91^^,^^113^^,^^117^^,^^124^^,^^129^^,^^137^^,^^148^^,^^150^^,^^151^ Other positions included the wrist (*n =* 5),^65^^,^^111^^,^^126^^,^^132^^,^^143^ arm (*n =* 3),^74^^,^^144^^,^^145^ trunk (*n =* 2),^118^^,^^122^ and thigh (*n =* 1),^75^ with 5 studies not reporting where the device was worn.^66^^,^^78^^,^^88^^,^^120^^,^^128^ Most studies that employed accelerometer-based measures used the ActiGraph (ActiGraph, LLC, Pensacola, FL, USA) GT3X+ (*n =* 4),^120^^,^^137^^,^^150^^,^^151^ wGT3X-BT (*n =* 2),^138^^,^^148^ or GT9X Link (*n =* 2).^78^^,^^132^ The other accelerometers were the RT3 (Stayhealthy Inc, Monrovia, CA, USA; *n =* 3),^117^^,^^118^^,^^124^ the SenseWear Pro3 Armband (BodyMedia, Pittsburgh, PA, USA; *n =* 3),^74^^,^^144^^,^^146^ the Activity Sensory Move II (movisens GmbH, Karlsruhe, Germany; *n =* 1),^129^ the LifeShirt (Vivometrics, Inc, Ventura, CA, USA; *n =* 1),^122^ the ActiWatch (Mini Mitter Co, Inc, Bend, OR, USA; *n =* 1),^111^ the AX3 (Axtivity, Newcastle upon Tyne, United Kingdom; *n =* 1),^65^ the FitBit (FitBit Inc, San Francisco, CA, USA) Charge HR (*n =* 1)^126^ and Charge 3 (*n =* 1),^143^ and the Activ8 (2 M Engineering, North Brabant, the Netherlands; *n =* 1).^66^ The type of accelerometer was not reported in 1 study.^113^ The pedometers were the Digi-Walker SW-200 (New Lifestyles Inc, Lees Summit, MO, USA; *n =* 1),^63^ the Active Style Pro HJA-350IT (Omron Heathcare, Kyoto, Japan; *n =* 1),^88^ and the Yamax Power-Walker EX-510 3D (Pedometer Express, Fertile, MN, USA; *n =* 1).^128^ These devices were worn for 5 days (*n =* 1),^111^ 6 days (*n =* 1^129^), 7 days (*n =* 16),^63^^,^^65^^,^^75^^,^^88^^,^^91^^,^^113^^,^^117^^,^^118^^,^^120^^,^^124^^,^^132^^,^^137^^,^^144^^,^^148^^,^^150^^,^^151^ or 14 days (*n =* 1).^126^ The remaining 7 studies did not specify the number of days the device was worn.^66^^,^^74^^,^^78^^,^^122^^,^^128^^,^^143^^,^^145^ All studies provided the accelerometer or pedometer on the day fear of movement was assessed (*n =* 18).^63^^,^^65^^,^^66^^,^^75^^,^^78^^,^^88^^,^^89^^,^^111^^,^^113^^,^^120^^,^^122^^,^^126^^,^^132^^,^^137^^,^^143^^,^^148^^,^^150^ The remaining studies did not specify whether fear of movement was measured on the day the device was provide or on the last day of physical activity assessment (*n =* 7).^74^^,^^118^^,^^124^^,^^128^^,^^144^^,^^146^^,^^151^

To assess physical activity, the studies used the following outcomes: score from a questionnaire (eg, Tegner Assessment Scale, Physical Activity Questionnaire for the Elderly, Baecke Habitual Physical Activity Questionnaire, Saltin-Grimby Physical Activity Level Scale, or Leisure Time Physical Activity Questionnaire; *n =* 24),^63^^,^^64^^,^^67^^,^^68^^,^^72^^,^^80^^,^^81^^,^^84^^,^^87^^,^^89^^,^^90^^,^^104^^,^^107^^,^^113^^,^^115^^,^^119^^,^^121^^,^^123^^,^^125^^,^^127^^,^^134^^,^^137^^,^^149^ metabolic equivalent task min/wk (*n =* 23),^31^^,^^73^^,^^76–79^^,^^83^^,^^85^^,^^86^^,^^112^^,^^114^^,^^116^^,^^130^^,^^131^^,^^133^^,^^136^^,^^138–142^^,^^146^^,^^147^ steps per day (*n =* 14),^63^^,^^65^^,^^75^^,^^78^^,^^88^^,^^113^^,^^120^^,^^126^^,^^128^^,^^132^^,^^143–145^^,^^150^ hours per day or week (*n =* 12),^62^^,^^65^^,^^69–71^^,^^74^^,^^91^^,^^113^^,^^120^^,^^138^^,^^143^^,^^145^ counts per minute (*n =* 4),^111^^,^^113^^,^^117^^,^^137^ kilocalories per day (*n =* 2),^129^^,^^144^ or percentage of active time (*n =* 1).^122^ Nine studies used multiple physical activity outcomes.^63^^,^^65^^,^^78^^,^^91^^,^^113^^,^^120^^,^^143–145^

#### Association Between Physical Activity and Fear of Movement

Among the 74 articles included in the systematic review, 42 reported correlation coefficients of the association between physical activity and fear of movement. Specifically, 32 articles reported at least 1 Pearson *r* correlation coefficient and 12 articles reported at least 1 Spearman ρ value.^87^^,^^89^^,^^91^^,^^113^^,^^116^^,^^124^^,^^127^^,^^132^^,^^135^^,^^143^^,^^149^^,^^151^ When a correlation coefficient was not reported but the exact *P* value (or *t* value) and sample size were available and it was possible to know the sign of the correlation—which was the case for 7 studies^83^^,^^111^^,^^115^^,^^120^^,^^125^^,^^133^^,^^144^—the Pearson *r* estimate was computed using an ad hoc R code (Suppl. Code 1A). For the studies that reported a relative *P* value of <.001 instead of an exact *P* value, we used a *P* value of .0009 to estimate an approximate *r* value.^82^

Through email correspondence with the authors, we obtained 23 additional Pearson *r* estimates.^29^^,^^62–81^^,^^104^^,^^107^ In total, 83 Pearson *r* estimates from 63 studies and 21 Spearman ρ estimates from 12 studies were used in the meta-analysis (Suppl. Table 1). The remaining study did not report a correlation coefficient and was therefore not included in the meta-analysis.^117^ This study reported a non–statistically significant positive association between physical activity and fear of movement based on a standardized β coefficient.

#### Pain

Mean pain intensity at rest was reported in 45 of the 74 articles included in the systematic review. Most studies used the visual analog scale^174^ (*n =* 21)^65^^,^^69–71^^,^^76^^,^^78^^,^^82^^,^^85^^,^^89^^,^^90^^,^^112^^,^^114^^,^^117^^,^^119^^,^^120^^,^^122^^,^^124^^,^^126^^,^^138^^,^^139^^,^^141^ or the numeric rating scale^175^ (*n =* 15).^29^^,^^62^^,^^66^^,^^68^^,^^75^^,^^79^^,^^80^^,^^83^^,^^88^^,^^111^^,^^113^^,^^116^^,^^127^^,^^128^^,^^137^ Other studies used the Knee Injury Osteoarthritis Outcome Score pain subscale^176^ (*n =* 3),^63^^,^^84^^,^^134^ the Brief Pain Inventory^177^ (*n =* 1),^64^ the Oxford Knee Score^178^ (*n =* 1),^140^ the Quality of Well-Being Scale–Self-Administered Pain Scale^179^ (*n =* 1),^150^ the 36-Item Short Form Health Survey for bodily pain^180^ (*n =* 1),^130^ the Graphic Rating Scale^181^ (*n =* 1),^133^ the Fibromyalgia Impact Questionnaire–Pain^182^ (*n =* 1),^67^ and the verbal rating scale^183^ (*n =* 1).^104^ In the meta-analysis, scores that were not on a scale from 0 to 100 in the initial measure were scaled to that range.

### Meta-Analysis

#### Main Meta-Analysis

**Figure 2:**
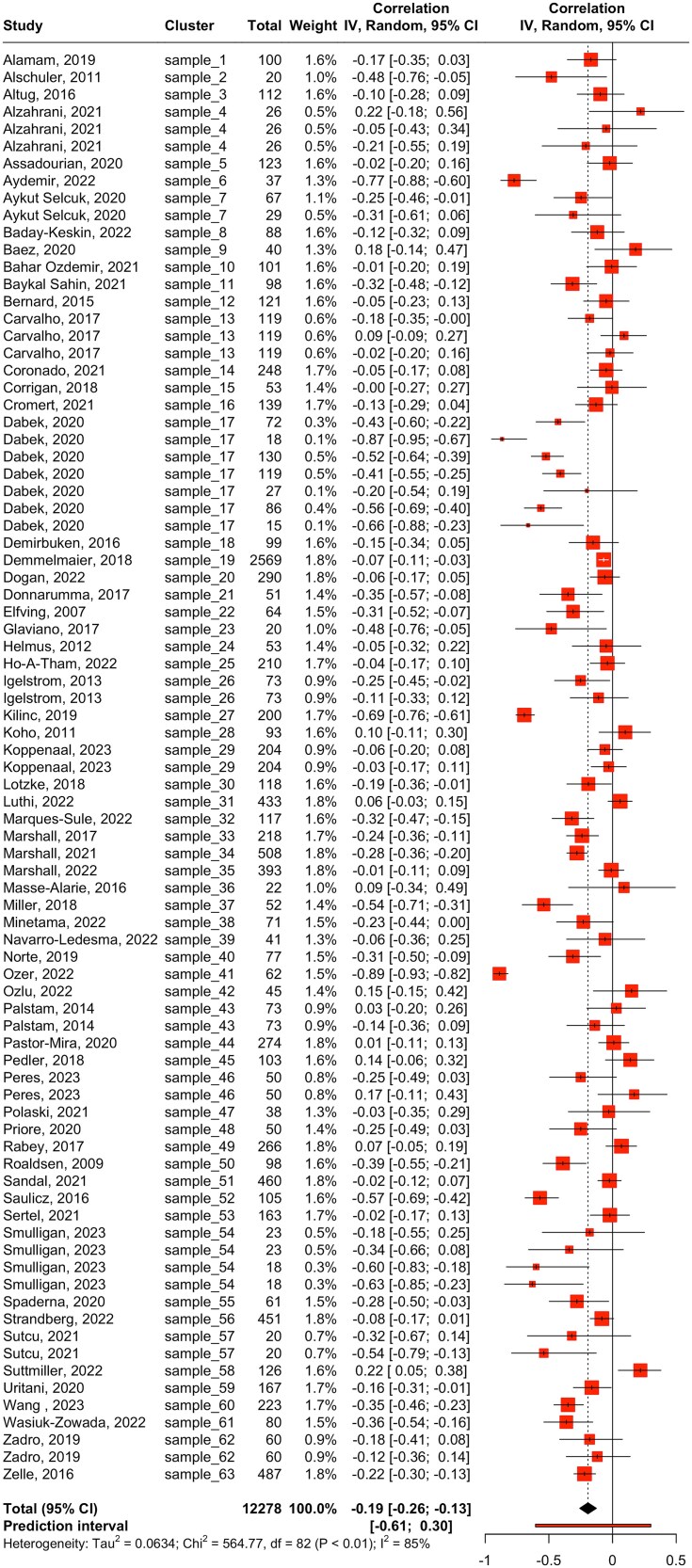
Main Meta-Analysis: Correlation Between Fear of Movement and Physical Activity. Abbreviations: IV = Inverse Variance Method; Random = Random-Effects Method.

Our main meta-analysis of 63 studies, 83 Pearson *r* correlation estimates, and 12,278 participants revealed a statistically significant negative correlation between fear of movement and physical activity (*r* = −0.19; 95% CI = −0.26 to −0.13; *P* < .0001) (Table 1; Figure 2). However, we observed substantial to considerable between-study statistical heterogeneity (τ^2^ = 0.06 [95% CI = 0.02 to 0.09]; *I*^2^ = 85.5% [95% CI = 82.6% to 87.9%]), and the prediction interval ranged from *r* = −0.605 to 0.300, indicating that a positive correlation cannot be ruled out for future studies.

**Table 1:** Results of the Main, Secondary, and Subgroup Meta-analyses^a^

Parameter	No. of Studies	No. of Estimates	No. of Observations or Participants	Correlation Estimate	95% CI	*I* ^2^ (%)	*P* *^b^*
Main: Pearson *r* estimates							<.0001
Fear of movement and physical activity	63	83	12,278	−0.19	−0.26 to −0.13	85	
Secondary: Spearman rho estimates							.0486
Fear of movement and physical activity	12	21	2084	−0.20	−0.38 to 0.001	86	
Subgroup: health status							<.0001
Chronic pain	29	35	5091	−0.07	−0.16 to 0.01	62	
Arthritis	9	11	3592	−0.25	−0.39 to −0.10	93	
Cardiovascular condition	5	10	823	−0.30	−0.47 to −0.11	29	
Neurologic condition	4	8	220	−0.53	−0.69 to −0.32	56	
Surgery	5	5	903	−0.16	−0.36 to 0.05	69	
Older adults	3	3	278	−0.40	−0.60 to −0.14	91	
Acute pain	2	2	103	−0.13	−0.29 to 0.04	34	
Pulmonary condition	2	2	285	−0.68	−0.82 to −0.46	98	
Obstructive sleep apnea	1	2	146	−0.18	−0.56 to 0.26	0	
Fibromyalgia	1	2	146	−0.06	−0.46 to 0.37	2	
Cancer	1	1	451	−0.19	−0.26 to −0.13		
Women postpartum	1	1	139	−0.13	−0.29 to −0.04		
Young adults	1	1	101	−0.01	−0.20 to 0.19		
Subgroup: physical activity measure							.1714
Self-reported	44	54	9882	−0.22	−0.29 to −0.14	89	
Device based	20	29	2396	−0.13	−0.24 to −0.02	57	
Subgroup: physical activity instrument							.2092
Accelerometer	18	27	2462	−0.17	−0.29 to −0.05	55	
IPAQ	18	25	5034	−0.28	−0.39 to −0.16	93	
BHPAQ	4	4	737	−0.34	−0.50 to −0.15	94	
SGPALS	4	4	675	−0.19	−0.43 to 0.08	80	
Ad hoc questionnaire	4	4	1240	−0.15	−0.39 to 0.11	85	
Pedometer	3	3	385	−0.02	−0.33 to 0.29	59	
GPAQ	2	2	232	0.01	−0.38 to 0.40	0	
MLTPAQ	2	2	580	−0.07	−0.42 to 0.29	87	
GLTEQ	2	2	118	−0.20	−0.54 to 0.20	43	
LTPAI	1	2	146	−0.06	−0.52 to 0.44	2	
SQUASH	1	2	100	−0.04	−0.52 to 0.46	77	
RAPAQ	1	2	120	−0.15	−0.59 to 0.30	0	
JPAS	1	1	126	0.22	0.05 to 0.38		
PASE	1	1	163	−0.02	−0.17 to 0.13		
UCLA	1	1	37	−0.77	−0.88 to −0.60		
Diary	1	1	123	−0.02	−0.20 to 0.16		
Subgroup: physical activity outcome							.6098
MET min/wk	25	35	6439	−0.20	−0.30 to −0.09	91	
Score	15	16	2090	−0.24	−0.36 to −0.11	85	
Steps/d	12	13	945	−0.18	−0.33 to −0.03	56	
Active time	10	12	2144	−0.11	−0.25 to 0.03	77	
Counts/min	5	5	579	−0.14	−0.33 to 0.05	8	
kcal/d	2	2	81	−0.38	−0.67 to 0.02	24	
Subgroup: fear of movement instrument							.3846
TSK-17	34	46	3799	−0.23	−0.31 to −0.14	86	
FABQ	13	15	5434	−0.13	−0.27 to 0.02	74	
TSK-11	8	8	1259	−0.04	−0.23 to 0.15	82	
TSK-Heart	1	7	467	−0.51	−0.78 to −0.08	61	
TSK-13	2	2	312	−0.17	−0.50 to 0.20	72	
TSK-14	1	1	451	−0.08	−0.17 to 0.01		
BBQ	1	1	223	−0.35	−0.46 to −0.23		
BFOMSO	1	1	167	−0.16	−0.31 to −0.01		
FActS	1	1	61	−0.28	−0.50 to −0.03		
KCS	1	1	105	−0.57	−0.69 to −0.42		

^a^ BBQ = Breathlessness Beliefs Questionnaire; BFOMSO = Brief Fear of Movement Scale for Osteoarthritis; BHPAQ = Baecke Habitual Physical Activity Questionnaire; FABQ = Fear Avoidance Beliefs Questionnaire; FActS = Fear of Activity in Situations; GLTEQ = Godin Leisure Time Exercise Questionnaire; GPAQ = Global Physical Activity Questionnaire; IPAQ = short form of the International Physical Activity Questionnaire; JPAS = Jurka Physical Activity Scale; KCS = Kinesiophobia Causes Scale; LTPAI = Leisure Time Physical Activity Index; MET = metabolic equivalent task; MLTPAQ = Minnesota Leisure Time Physical Activity Questionnaire; PASE = Physical Activity Scale for the Elderly; RAPAQ = Rapid Assessment of Physical Activity Questionnaire; SGPALS = Saltin-Grimby Physical Activity Level Scale; SQUASH = Short Questionnaire to Assess Health Enhancing Physical Activity; TSK = Tampa Scale for Kinesiophobia (adaptation of the TSK for patients with coronary artery disease [TSK-Heart]; 11-item TSK [TSK-11]; 13-item TSK [TSK-13]; 14-item TSK [TSK-14]; 17-item TSK [TSK-17]); UCLA = University of California–Los Angeles activity score.

^b^ *P* value for between-group difference.

The sampling error variance on level 1 and the value of *I*^2^ on level 2, that is, the amount of heterogeneity variance within studies, were small (10.3% and 8.2%, respectively). The largest share of heterogeneity variance was from level 3, with between-study heterogeneity making up 81.5% of the total variation in our data (Suppl. Figure 1). Overall, this indicates that there is considerable between-study heterogeneity, and less than one-tenth of the variance can be explained by differences within studies.

The 3-level model showed a better fit than the 2-level model with lower Akaike information criterion (28.4 vs 39.0) and Bayesian information criterion (35.6 vs 43.8) values, indicating better performance. These lower Akaike information criterion and Bayesian information criterion are consistent with the significant likelihood ratio test comparing the 2 models (χ^2^ = 12.67; *P* = .0004). Therefore, although the 3-level model introduces an additional parameter, this added complexity has improved our estimate of the pooled effect.

#### Publication Bias Assessment

**Figure 3:**
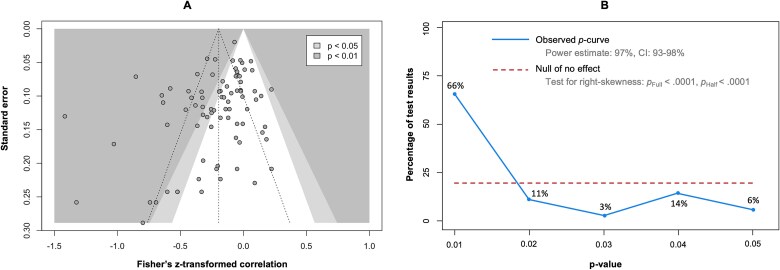
Publication Bias Assessment. (A) Contour-enhanced funnel plot of the main meta-analysis. The vertical dashed line represents the average effect size. The 2 other dashed lines represent the idealized funnel shape that studies are expected to follow. (B) *p*-curve analysis. The blue line indicates the distribution of the analyzed *P* values. The red dotted line illustrates a uniform distribution of the *P*, indicating the absence of a true effect. *p*_Full_ = *P* value from the right-skewness test using all significant *P* (*P* < .05); *p*_Half_ = *P* value from the right-skewness test using only the most significant *P* (*P* ≤ .025).

The Egger regression test using the SEs of the effect size estimates as a predictor in the meta-regression showed that the coefficient of the SE was significant (β [*b*] = −1.497 [95% CI = −2.618 to −0.3754]; *P* = .0095), suggesting that the data in the funnel plot were asymmetrical (Figure 3A). This asymmetry may be explained not only by publication bias but also by other potential causes, such as different study procedures and between-study heterogeneity,^184^ which was substantial to considerable here.

The 83 Pearson *r* correlation estimates were provided to the *p*-curve analysis. The observed *p*-curve included 35 statistically significant results (*P* < .05), 27 of which were highly significant (*P* < .025), and were visually right skewed (Figure 3B). The other results were excluded because they had a *P* value of >.05. The *P* value of the right skewness test was <.001 for both the half curve (curve of *P* values of ≤ .025) and the full curve (curve of *P* values of < .05), confirming that the *p*-curve was right skewed and suggesting that the effect of our meta-analysis is true—namely, that the effect we estimated is not an artifact caused by selective reporting (eg, *p*-hacking) in the literature.^185^ In addition, the statistical power of the studies that were included in the *p*-curve analysis was 97% (90% CI = 93% to 98%), suggesting that approximately 90% of the significant results are expected to be replicable.

#### Secondary Meta-Analysis

Results of the secondary meta-analysis of 12 studies, 21 Spearman ρ correlation estimates, and 2084 participants were consistent with the main meta-analysis as it showed a statistically significant negative correlation between fear of movement and physical activity (*r* = −0.20 [95% CI = −0.38 to −0.01]; *P* = .049) (Table 1; Suppl. Figure 2). However, we observed substantial to considerable between-study statistical heterogeneity (τ^2^ = 0.10 [95% CI = 0.04 to 0.28]; *I*^2^ = 86.3%) and the prediction interval ranged from *r* = −0.710 to 0.445, indicating that a positive correlation cannot be ruled out for future studies.

#### Subgroup Meta-Analyses

**Figure 4:**
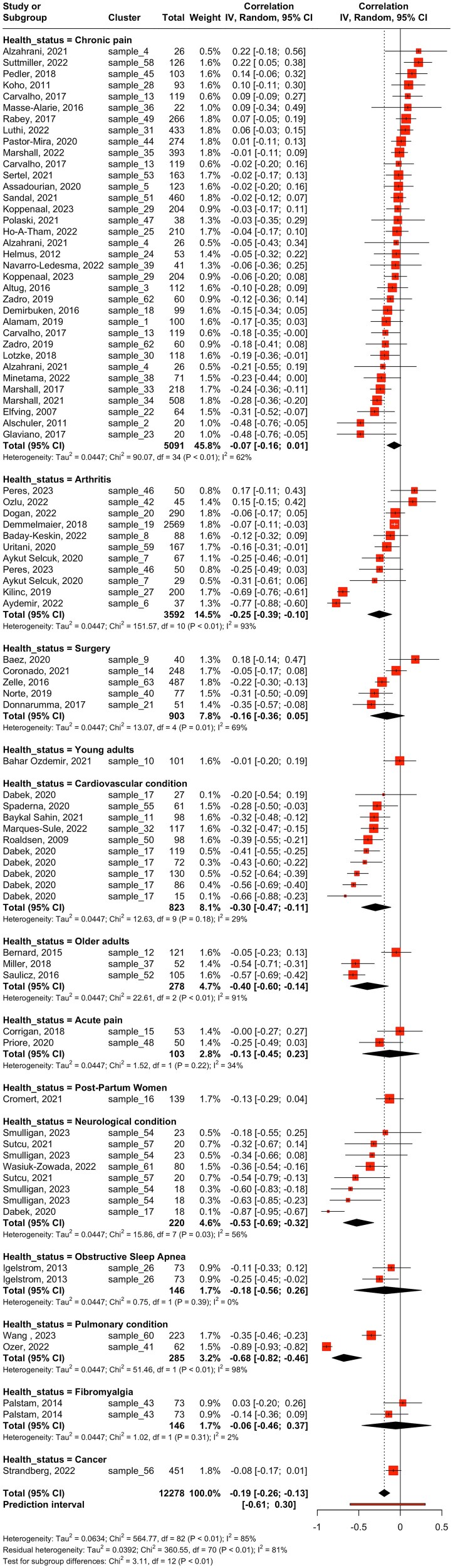
Subgroup Meta-Analysis: Differences According to Health Status. Abbreviations: IV = Inverse Variance Method; Random = Random-Effects Method.

The test of subgroup differences between health status was conducted on studies comprising people with chronic (number of studies [*k*] = 35) or acute pain (*k* = 2), arthritis (*k* = 11), a cardiovascular condition (*k* = 10), a neurologic condition (*k* = 8), surgery (*k* = 5), older age (*k* = 3), obstructive sleep apnea (*k* = 2), a pulmonary condition (*k* = 2), fibromyalgia (*k* = 2), or cancer (*k* = 1), as well as women postpartum (*k* = 1) and young adults who were healthy (*k* = 1) (Table 1; Figure 4). We found a statistical moderating effect of health status (*P* = .0014). The relationship between fear of movement and physical activity was statistically significant only in studies that included participants with a cardiac condition (*r* = −0.30 [95% CI = −0.47 to −0.11]), arthritis (*r* = −0.25 [95% CI = −0.39 to −0.10]), a neurologic condition (*r* = −0.53 [95% CI = −0.69 to −0.32]), or a pulmonary condition (*r* = −0.68 [95% CI = −0.82 to −0.46]) or older adults (*r* = −0.40 [95% CI = −0.60 to −0.14]). We found no evidence of an association between fear of movement and physical activity in studies that included participants with chronic pain (*r* = −0.07 [95% CI = −0.16 to 0.01]) or acute pain (*r* = −0.13 [95% CI = −0.45 to 0.23]). Statistical heterogeneity was higher in the studies comprising people with a pulmonary condition (*I*^2^ = 98.1%), people with arthritis (*I*^2^ = 93.4%), or older adults (*I*^2^ = 91.2%) than in the studies comprising people with a cardiac (*I*^2^ = 28.7%) or neurologic (*I*^2^ = 55.9%) condition.

**Figure 5:**
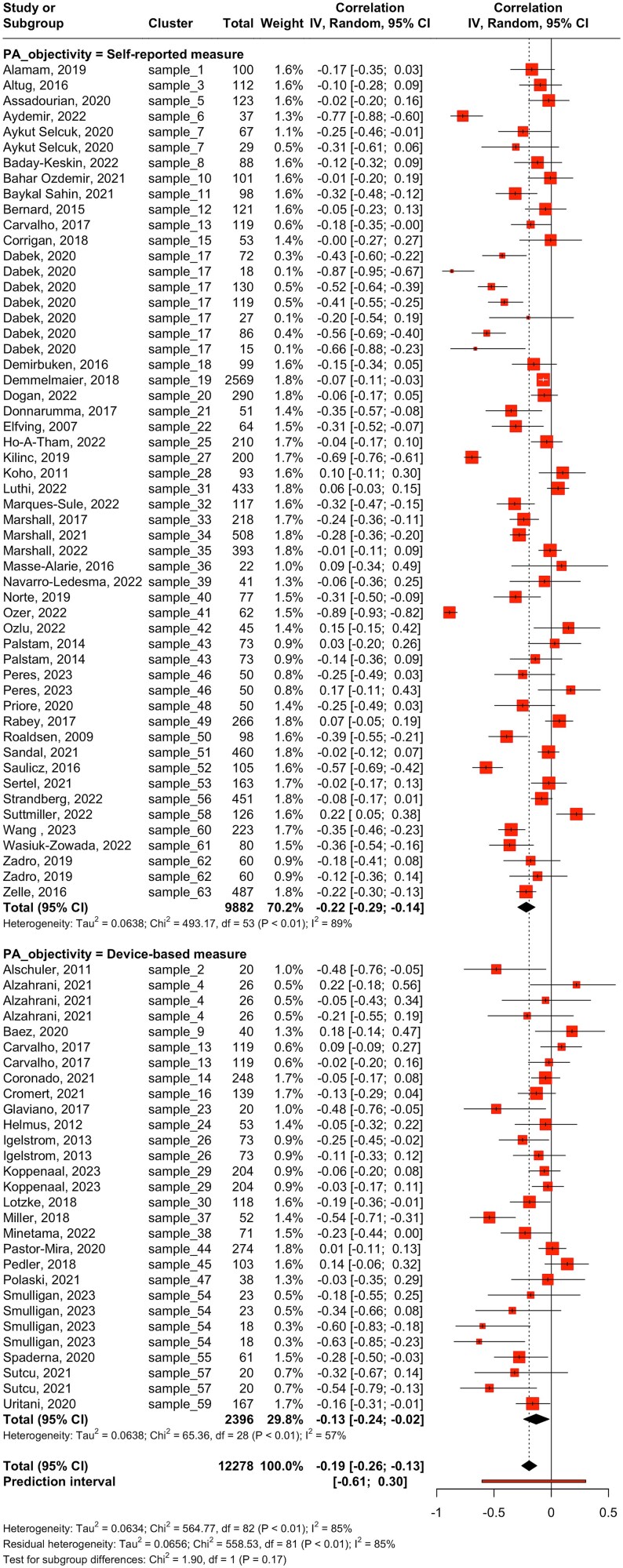
Subgroup Meta-analysis: Differences According to Physical Activity (PA) Measure (Self-Reported vs Device Based). Abbreviations: IV = Inverse Variance Method; Random = Random-Effects Method.

The test of subgroup differences between self-reported (*k* = 54) and device-based (*k* = 29) measures of physical activity showed no evidence of a moderating effect of the type of physical activity measure (*P* = .171) (Table 1). Both self-reported measures (*r* = −0.22 [95% CI = −0.29 to −0.14]; *I*^2^ = 89.3%) and device-based measures (*r* = −0.13 [95% CI = −0.24 to −0.02]; *I*^2^ = 57.2%) (Figure 5) showed a negative association between fear of movement and physical activity.

We also found no evidence suggesting that physical activity instruments (*P* = .209) (Suppl. Figure 3), physical activity outcome (*P* = .685) (Suppl. Figure 4), or fear of movement instrument (*P* = .385) (Suppl. Figure 5) moderated the relationship between fear of movement and physical activity.

#### Meta-Regressions

Age did not statistically influence the correlation estimates of the meta-analysis studies (*k* = 72; *P* = .349) (Suppl. Figure 6A). Similarly, the proportion of women (*k* = 72; *P* = .555) (Suppl. Figure 6B) and the mean level of pain in the studies (*k* = 49; *P* = .481) (Suppl. Figure 6C) did not influence correlation estimates.

#### Sensitivity Analysis

The meta-regression by quality score showed that a study’s quality did not influence correlation estimates (*k* = 83; *P* = .373).

## DISCUSSION

The main objective of this study was to systematically review and meta-analyze the direct relationship between fear of movement and physical activity. In addition, we examined the influence of potential moderators, such as health status. To our knowledge, this is the first review of its kind on this research topic.

### Fear of Movement as a Barrier to Physical Activity

Both the main meta-analysis based on Pearson *r* correlation estimates and the secondary meta-analysis based on Spearman ρ correlation estimates showed a negative correlation between fear of movement and physical activity. These results indicate that individuals with higher fear of movement are likely to engage in less physical activity, highlighting the importance of assessing fear of movement in clinical practice, as it may affect participation in exercise and rehabilitation programs. Rehabilitation professionals should be vigilant in identifying and assessing patients who may be experiencing fear of movement, as it may impede their recovery or ability to maintain a healthy lifestyle.

These results are consistent with our hypothesis and the dual models of physical activity.^32–35^ According to these theoretical models, our findings suggest that fear of movement triggers an impulse to avoid physical activity behaviors, which contributes to the maintenance or exacerbation of the initial fear. Thus, fear of movement and physical inactivity can constitute a self-perpetuating or even self-reinforcing cycle.

### Patient-Specific Interventions

The subgroup analysis reveals that the negative relationship between fear of movement and physical activity is more pronounced in certain populations, including those with a cardiac condition, a neurologic condition, a pulmonary condition, or arthritis, as well as older adults. This result suggests that fear of movement should be included in the management plans of these populations. Specifically, physical therapists, in collaboration with other health professionals, may need to address not only the physical aspects of rehabilitation but also the psychological components related to the fear of movement. Cognitive behavioral therapy,^186^ graded exposure therapy,^187^ or psychoeducation^188^ are potential approaches to consider for helping patients overcome this fear. Indeed, multidisciplinary interventions including psychological treatment as well as physical therapy exercises have been shown to successfully reduce fear of movement.^39^

In individuals with a cardiac condition, fear of movement and its association with physical activity may be explained by concerns about triggering another cardiac event^189^ or worsening their condition.^190^ Breathlessness (dyspnea) further reduces the ability to be physically active and damages confidence, which leads to persistent anticipation of negative outcomes from physical activity.^191^ Dyspnea is also a major barrier to physical activity in people with a pulmonary condition, such as chronic obstructive pulmonary disease (COPD).^147^ Patients with asthma face additional barriers, including the fear of provoking respiratory symptoms and exacerbations.^192^ Chest tightness commonly experienced in patients with Parkinson disease may contribute to the fear of movement and its impact on physical activity.^193^ Similarly, the fear of falling may be an explanatory factor in patients with Parkinson disease,^193^ people who have survived stroke,^194^ and older adults who are healthy.^195^ In patients with osteoarthritis, the belief that physical activity will “damage the joints”^196^ and the perceived fragility of their physical status^197^ may also contribute to this relationship.

Although our results showed no evidence of an association between fear of movement and physical activity in other health conditions, such as cancer, postsurgery status, postpartum status, or obstructive sleep apnea, these effects cannot be fully ruled out, as the lack of statistical significance could be explained by a lack of statistical power in these subgroup meta-analyses including fewer estimates (*k* = 1 to 5).

### Pain Versus Fear

Our results showed no evidence of an association between fear of movement and physical activity in people with fibromyalgia, acute pain, or chronic pain. This finding was surprising because fear of pain is a key component of fear of movement assessment, appearing in 10 of the 17 items on the TSK-17 and TSK-Heart scales, and reinforces the importance of considering the multidimensional nature of fear of movement, which not only relates to pain but also reflects fear of injury and fear of worsening a health condition. Because pain could have influenced the results in other health conditions, we conducted a meta-regression analysis including all studies that assessed pain, irrespective of health condition. Again, results showed no statistical evidence suggesting that pain intensity at rest influenced the effect of fear of movement on physical activity, despite the substantial number of estimates included in this analysis (*k* = 49). These results are consistent with the weak relationship that has previously been reported between fear of movement and pain.^198^ These results suggest that in these populations, other factors, such as psychological distress and actual pain intensity, may play a more prominent role than fear of movement in influencing physical activity levels.

However, this absence of evidence might be related to the methods used to assess pain, which may be better assessed by pain history (eg, pain duration in months) or pain intensity during exercise. Another explanation may be that the assessments of physical activity in the included studies focused on differences in intensity but did not differentiate between types of activities (eg, aerobic activities, strength training, stretching, balance activities). The relationship between fear of movement and physical activity may depend on the type of physical activity. Consistent with this potential explanation, studies of chronic pain suggest that physical activity is not always avoided altogether and that avoidance behaviors may be more subtle.^199^ For example, some physical activities, such as strength training, may be fear-inducing in this population, whereas walking may not be because it is known to be painless and to improve spinal health. Therefore, future studies should examine whether the relationship between fear of movement and physical activity is moderated by the type of physical activity.

### Self-Report Versus Device-Based Measures of Physical Activity

Importantly, the negative correlation between fear of movement and physical activity was observed both in studies using self-report (eg, IPAQ) and device-based measures of physical activity (ie, accelerometers or pedometers), suggesting that they are similarly effective in capturing the relationship between fear of movement and physical activity. Self-report and device-based measures of physical activity have shown low to moderate correlations,^200^ suggesting that they provide related but distinct information. The information assessed by both measures is the level of physical activity; the distinct information is related to factors that specifically influence the self-report measure, such as social desirability, quality of life, well-being, and social support.^201^^,^^202^ Although device-based measures of physical activity are more valid than self-reports, our results suggest that the latter provide a sufficiently valid measure of physical activity for assessing its relationship with fear of movement.

### Limitations

The results of this systematic review and meta-analysis should be interpreted with consideration of several limitations. First, the considerable heterogeneity across the included studies may be explained not only by the diversity of the methods used to assess physical activity (questionnaires vs accelerometers vs pedometers), the instruments used in these methods (different questionnaires; different accelerometers and pedometers), and the physical activity outcomes but also by the different questionnaires used to assess fear of movement. Second, because fear of movement is a state, that is, a dynamic psychological variable, the time difference between the physical activity and fear of movement assessments, as well as the context of assessment, may have influenced the results. Third, although a subgroup meta-analysis showed no evidence of an effect of the type of TSK scale, inconsistencies have been noted in the purported dimensions assessed by different TSK scales or across populations,^203^ which may have influenced our results. Fourth, only 21 of the 98 authors we contacted (21%) shared their estimates (*n =* 13) or raw data (*n =* 8) with us, which is more than reported in previous literature.^204^ Including these missing data may have affected the results.

## CONCLUSIONS

Higher levels of fear of movement were associated with lower levels of physical activity, especially in people with a cardiac, neurologic, arthritic, or pulmonary condition. According to theoretical models, this relationship between fear of movement and physical activity results from automatic processes that may be self-reinforcing and should therefore not be overlooked. However, heterogeneity between studies was substantial to considerable for some results, and the evidence for publication bias calls for cautious conclusions about this potential relationship. More evidence is required to determine the impact fear of movement should have on therapeutic decisions when aiming to maintain or increase physical activity. Particularly, prospective studies are needed to better understand the factors and mechanisms that influence the relationship between fear of movement and physical activity.

This study underscores the importance of integrating psychological care into physical rehabilitation, particularly for patient populations for whom fear of movement is a significant barrier to recovery. Rehabilitation professionals should be trained to recognize psychological barriers and be aware of evidence-based interventions that can modify maladaptive beliefs about movement, ultimately promoting more active lifestyles.

## Supplementary Material

2024_0387_R1_supplemental_PTJ_2024_0387_final_pzaf050

## Data Availability

According to good research practices,^44^ the dataset, R Markdown script, and supplementary material are freely available in Zenodo.^206^ A preprint version of this manuscript is publicly available online^205^ and has been recommended by Peer Community In (PCI) Health & Movement Sciences.^207^

## SYSTEMATIC REVIEW REGISTRATION

To reduce literature bias,^43^^,^^44^ this systematic review was preregistered at the International Prospective Register of Systematic Reviews (PROSPERO; registration number CRD42022364063).^45^
