# Supplementary material for: Relationship Between Fear of Movement and Physical Activity in Patients With Cardiac, Rheumatologic, Neurologic, Pulmonary, or Pain Conditions: A Systematic Review and Meta-Analysis
Source: Phys Ther. 2025 Apr 6;105(6):pzaf050. doi: 10.1093/ptj/pzaf050 (PMC12207065; doi:10.1093/ptj/pzaf050)
Supplement: 2024_0387_R1_supplemental_PTJ_2024_0387_final_pzaf050 [file 2024_0387_r1_supplemental_ptj_2024_0387_final_pzaf050.pdf]

SUPPLEMENTARY MATERIAL

Relationship between fear of movement and physical activity in patients with cardiac, rheumatologic, neurologic, pulmonary, or pain conditions: A systematic review and meta-analysis

Miriam Goubran[1,2,†], Ata Farajzadeh[1,2,†], Ian M. Lahart[3], Martin Bilodeau[1,2], & Matthieu P. Boisgontier[1,2,4,*]

[1]School of Rehabilitation Sciences, Faculty of Health Sciences, University of Ottawa, Canada; [2]Bruyère Health Research Institute, Ottawa, Canada; [3]Faculty of Education, Health and Wellbeing, Institute of Human Sciences, University of Wolverhampton, Wolverhampton, UK; [4]Perley Health Centre of Excellence in Frailty-Informed Care, Ottawa, Canada. [†]MG and AF contributed equally to this work. *Corresponding author: matthieu.boisgontier@uottawa.ca

Supplementary Code 1. R scripts.

Supplementary Table. Sample characteristics of studies included in the systematic review.

Supplementary Figure 1. Heterogeneity variance.

Supplementary Figure 2. Secondary meta-analysis based on Pearson’s rho estimates.

Supplementary Figure 3. Subgroup meta-analysis: Differences by physical activity measurement instrument.

Supplementary Figure 4. Subgroup meta-analysis: Differences by physical activity outcome

Supplementary Figure 5. Subgroup meta-analysis: Differences by fear of movement measurement instrument.

Supplementary Figure 6. Meta-regressions testing the influence of age (A; 72 studies), the proportion of women (B; 72 studies), and pain intensity (C; 49 studies) on the relationship between fear of movement and physical activity.

Supplementary Code 1. R scripts.

A. R script for the calculation of Pearson's r estimate based on the degrees of freedom (sample size - 2) and exact p-value, when the direction of the relationship is known.

```
# Sample size
n <- 50

# Pearson correlation coefficient
r <- -0.57

# Compute the t-statistic
t_statistic <- r * sqrt((n - 2) / (1 - r^2))

# Degrees of freedom
df <- n - 2

# Compute the p-value
p_value <- 2 * pt(abs(t_statistic), df = df, lower.tail = FALSE)

# Print the p-value
print(p_value)
```

B. R script for the calculation of exact p-values based on sample size (n) and Pearson's correlation coefficient (r).

```
# Sample size
n <- 45

# Pearson correlation coefficient
r <- -0.25

# Compute the t-statistic
t_statistic <- r * sqrt((n - 2) / (1 - r^2))

# Degrees of freedom
df <- n - 2

# Compute the p-value
p_value <- 2 * pt(abs(t_statistic), df = df, lower.tail = FALSE)

# Print the p-value
print(p_value)
```

Supplementary Table. Sample Characteristics of Studies Included in the Systematic Review[a]

Study	No. of Participants (No. of Women)	Mean Age (SD or Range)	Health Status or Population	Fear of Movement		Physical Activity		Level of Pain		Study Design	Context of Assessment	Normalized Quality Score	Correlation	*P*
				Mean (SD or Range)	Measure	Mean (SD or Range)	Tool	Mean (SD)	Tool					
Alamam et al[76] (2019)	100 (62)	40 (13.6)	Chronic low back pain	n.a.	FABQ	n.a.	IPAQ	4.8	VAS	Cohort	Clinical	7.0	$r = -0.17$	.116
Alzahrani et al[65] (2021)	26 (11)	43.6 (14.3)	Chronic low back pain	40.19 (9.20)	TSK-17	76.07 min of MPA/d (34.45)	Wrist accelerometer	4 (3)	VAS	RCT	Clinical	8.8	$r = 0.22$	.28[c]
						0.29 min of VPA/d (1.22)	Wrist accelerometer						$r = -0.05$	.003[c]
						13,302 steps/d (5141)	Wrist accelerometer						$r = -0.21$	.30[c]
Alschuler et al[111] (2011)	20 (9)	46.1 (9.35)	Chronic low back pain	30.55	TSK-17	228 counts/min	Wrist accelerometer	4.87	NRS	Cross-sectional	Clinical	6.0	$r = -0.48$[b]	.03
Altuğ et al[112] (2016)	112 (73)	45.0 (14.6)	Chronic low back pain	44.30	TSK-17	5495 MET min/wk	IPAQ	3.45	VAS	Cross-sectional	Clinical	8.0	$r = -0.096$	.313
Assadourian et al[62] (2020)	147 (88)	49 (12)	Chronic low back pain	n.a.	TSK-17	n.a.	Diary (<1 vs ≥1 h/wk)	6.7	NRS	Cross-sectional	n.a.	8.0	$r = -0.022$[d]	.813[d]
Atici et al[149] (2022)	254 (171)	n.a. (>65)	Older adults	54.55	KCS	182.8	PASE	n.a.		Cross-sectional	Online	9.0	$\rho = -0.345$	<.001
Aydemir et al[84] (2022)	37 (25)	58.8 (8.6)	Knee osteoarthritis	40.3	TSK-17	4.8	UCLA	52.0	KOOS-P	Cross-sectional	Clinical	7.0	$r = -0.773$	<.05

Aykut Selçuk and Karakoyun[138] (2020)	67 (67)	60.6 (8.0)	Knee osteoarthritis	44.8	TSK-17	n.a.	IPAQ (low vs moderate vs high)	4.6	VAS	Cross-sectional	Clinical	7.0	$r = -0.247$	.019
	29 (0)	61.6 (8.1)	Knee osteoarthritis	42.0	TSK-17	n.a.	IPAQ (low vs moderate vs high)	2.9	VAS				$r = -0.309$	.116
Baday-Keskin and Ekinci[85] (2022)	88 (67)	52 (n.a.)	Rheumatoid arthritis	45	TSK-17	594 MET min/wk	IPAQ	4.8	VAS	Cross-sectional	Clinical	7.0	$r = -0.12$	>.05
	93 (67)	45 (n.a.)	Adults who are healthy	39	TSK-17	971 MET min/wk	IPAQ	n.a.					n.a.	
Baez et al[63] (2020)	40 (24)	24.3 (4.1)	Surgery (ACLR)	18.2	TSK-11	8657 steps/d	Hip pedometer	81.5	KOOS-P	Cross-sectional	Clinical	8.0	$r = 0.181$[d]	.265[d]
						7.7	TAS						n.a.	n.a.
Bahar Özdemir[29] (2021)	101 (59)	33.9 (6.0)	People who are healthy	36.4 (5.8)	TSK-17	756 MET min/wk (1090)	IPAQ	3.1 (3.3)	NRS	Cross-sectional	Online	8.0	$r = -0.007$[d]	.944[d]
Barchek et al[132] (2021)	19 (13)	22.9 (3.2)	Anterior cruciate ligament reconstruction	6 (8)	FABQ	11,237.7 steps/d (5667.7)	Wrist accelerometer	n.a.		Cross-sectional	Clinical	7.0	ρ for steps/d = 0.12	.63
													ρ for counts/min = −0.13	.59
Baykal Şahin et al[130] (2021)	98 (35)	58.1 (10.4)	Coronary artery disease	41.4 (6.2)	TSK-17	839 MET min/wk (1212)	IPAQ	60.1 (27.2)	SF-36	Cross-sectional	Clinical	8.0	$r = -0.315$	.002
Bernard et al[72] (2015)	121 (121)	65.5 (57–75)	Women who are postmenopausal	36	TSK-17	n.a.	PAQE	n.a.		RCT	Clinical	8.8	$r = -0.05$[d]	.55[d]
Carvalho et al[113] (2017)	119 (82)	39.1 (11.2)	Chronic low back pain	41	TSK-17	6844 steps/d	Hip accelerometer	6.7	NRS	Cross-sectional	Clinical	9.0	ρ = −0.15	>.05

						296 counts/min	Hip accelerometer						$r = -0.02$	>.05
						22 min of MVPA/d	Hip accelerometer						$\rho = -0.13$	>.05
						333 min of LPA/d	Hip accelerometer						$r = 0.09$	>.05
						6.7	BHPAQ						$r = -0.18$	<.05
Coronado et al[137] (2021)	248 (126)	62.2 (11.9)	Surgery (laminectomy)	28.4	TSK-13	427 counts/min	Hip accelerometer	3.1	NRS	Cohort	n.a.	8.0	$r = -0.05$	>.05
Corrigan et al[125] (2018)	53 (18)	54.8 (34–65)	Achilles tendinopathy	35.4	TSK-17	n.a.	SGPALS	n.a.		Cross-sectional	Clinical	8.0	$r = -0.005$[b]	.969
Crommert et al[148] (2021)	139 (139)	37 (4.9)	Postpartum disability	n.a.	TSK-17	n.a.	Hip accelerometer	n.a.		Cross-sectional	Online	7.0	$r = -0.13$	>.05
Dąbek et al[86] (2020)	130 (n.a)	n.a.	Coronary disease	44.3	TSK-Heart	1545 MET min/wk	IPAQ	n.a.		Cross-sectional	Clinical	8.0	$r = -0.523$	<.001
	119 (n.a)	n.a.	Hypertension	44.4	TSK-Heart	1509 MET min/wk	IPAQ						$r = -0.410$	<.001
	27 (n.a)	n.a.	Heart valve defect	44.4	TSK-Heart	1308 MET min/wk	IPAQ						$r = -0.201$	>.05
	72 (n.a)	n.a.	Myocardial infarction	46.7	TSK-Heart	1369 MET min/wk	IPAQ						$r = -0.428$	<.001
	86 (n.a)	n.a.	Rhythm disorder	43.3	TSK-Heart	1660 MET min/wk	IPAQ						$r = -0.563$	<.001

	18 (n.a)	n.a.	Stroke	49.7	TSK-Heart	1135 MET min/wk	IPAQ						$r = -0.868$	<.001
	15 (n.a)	n.a.	Other CVD	44.2	TSK-Heart	2207 MET min/wk	IPAQ						$r = -0.663$	.01
Demmelmaier et al[82] (2018)	2569 (1875)	60 (11)	Rheumatoid arthritis	n.a.	FABQ	n.a.	IPAQ	3.2	VAS	Cross-sectional	Online	9.0	$r = -0.07$[b]	<.0001
Demirbüken et al[114] (2016)	99 (65)	43.5 (12.8)	Chronic neck pain	41.82	TSK-17	3749 MET min/wk	IPAQ	6.47	VAS	Cross-sectional	Clinical	8.0	$r = -0.153$	.13[c]
Doğan and Taşci[139] (2022)	290 (178)	59.86 (15.64)	Knee osteoarthritis	45.3 (8.6)	TSK-17	n.a.	IPAQ	5.23 (1.52)	VAS	Cross-sectional	Clinical	8.0	$r = -0.061$	.299
Donnarumma et al[133] (2017)	51 (12)	61.9 (13.9)	Surgery (laminectomy)	n.a.	TSK-17	n.a.	IPAQ	3.5	GRS	Cohort	Clinical	7.0	$r = -0.35$[b]	.01
Elfving et al[115] (2007)	64 (39)	47 (19–64)	Chronic low back pain	n.a.	TSK-13	n.a.	SGPALS	n.a.		Cross-sectional	Online	9.0	$r = -0.31$[b]	.010
Glaviano et al[126] (2017)	20 (15)	22.2 (2.6)	Patellofemoral pain	13.6 (4.4)	FABQ	8629.7 steps/d (1665.3)	Wrist accelerometer	4.4 (1.9)	VAS	Cross-sectional	Clinical	8.0	$r = -0.481$	.02
González de la Flor et al[116] (2022)	42 (32)	36.7 (13.2)	Chronic headache	9	TSK-11	n.a.	IPAQ	7.14	NRS	Cross-sectional	n.a.	8.0	$\rho = 0.204$	.20[c]
Helmus et al[117] (2012)	53 (37)	39.9 (11.3)	Chronic MSK pain	35.4	TSK-17	138 counts/min	Hip accelerometer	5.8	VAS	Cross-sectional	Clinical	8.0	$r = -0.05$	.75
Ho-A-Tham et al[83] (2022)	210 (210)	48.7 (16.9)	Chronic low back pain	n.a.	FABQ	2240 MET min/wk	GPAQ	n.a.	NRS	Cross-sectional	Home visit	8.0	$r = -0.04$[b]	.49
Huijnen et al[118] (2010)	111 (52)	44.1 (10.3)	Subacute low back pain	36.0	TSK-17	n.a.	Trunk accelerometer	n.a.	NRS	Cohort	Clinical	7.0	$\beta = 0.12$	>.05

Igelström et al[145] (2013)	63 (15)	55 (12)	Obstructive sleep apnea syndrome	12.4 (3.1)	TSK-7	7734 steps (3528)	Armband accelerometer	n.a.		Cross-sectional	Clinical	8.0	*r* = −0.251	.049
						77 min of MVPA (54)	Armband accelerometer						*r* = −0.115	.37
Kilinç et al[140] (2019)	200 (120)	53.2 (6.0)	Knee osteoarthritis	31.8	TSK-17	1947 MET min/wk	IPAQ	24.1	OKS	Cross-sectional	Clinical	8.0	*r* = −0.693	<.001
Knapik et al[87] (2019)	135 (59)	71.9 (4.8)	Coronary artery disease	43	TSK-Heart	2.60	Ad hoc questionnaire	n.a.		Cross-sectional	Clinical	8.0	ρ = −0.8	2.6×10^{-35}[c]
Koppenaal et al[66] (2023)	204 (102)	47.68 (49.05)	Chronic low back pain	26.5 (16.1)	FABQ	77.58 min/d (38.85)	Accelerometer	5.3 (2)	NRS	RCT	Clinical	9.2	*r* for MPA = −0.06	.39
													r for VPA = −0.03	.69
Koho et al[119] (2011)	93 (60)	44.0 (17–68)	Chronic pain	n.a.	TSK-17	n.a.	LTPAQ	6.4	VAS	Cohort	Clinical	7.0	*r* = 0.10	>.05
Leonhardt et al[127] (2009)	449 (224)	45.4 (12.3)	Acute back pain	17	FABQ	34.7	FQPA	4.9 (1.7)	NRS	Cohort	Clinical	7.0	ρ = −0.93	<.05
	338 (17)	50.4 (13.2)	Chronic back pain	18.4	FABQ	44.1	FQPA						ρ = −0.95	>.05
Lotzke et al[120] (2018)	118 (63)	46 (8)	Chronic low back pain	38.1	TSK-17	198 min of MVPA/wk	Accelerometer	6.1	VAS	Cross-Sectional	Clinical	9.0	n.a.	n.a.
						7493.5 steps/d	Accelerometer						*r* = −0.19[b]	.034
Luthi et al[64] (2018)	433 (n.a.)	n.a.	Chronic MSK pain	44.6	TSK-17	4.45	BHPAQ	4.45	BPI-S	Cross-Sectional	Clinical	8.0	*r* = 0.067[d]	.759[d]
Marques-Sule et al[131] (2022)	117 (51)	56 (12.1)	Heart transplantation	32.5	TSK-11	219 MET min/wk	IPAQ	n.a.		Cross-sectional	Clinical	9.0	*r* = −0.32	.001

Marshall et al[69] (2017)	218 (130)	36.3 (6.6)	Chronic low back pain	13.8 (5.6)	FABQ	n.a.	Open-ended questions (<30 vs ≥30 min/wk)	3.6 (2.3)	VAS	Cross-sectional	Clinical	8.0	r = −0.24	.0003[b]
Marshall et al[70] (2021)	508 (259)	38.2 (11.8)	Chronic low back pain	13.2 (5.7)	FABQ	n.a.	Open-ended questions (<30 vs ≥30 min/wk)	4.5 (2.5)	VAS	Cross-sectional	Clinical	8.0	r = −0.28	1.3×10^{-10}[b]
Marshall et al[71] (2022)	393 (193)	39.2 (12.2)	Chronic low back pain	13.3 (5.2)	FABQ	n.a.	AHS-derived questions (<30 vs ≥30 min/wk)	4.3 (2.3)	VAS	Cohort	Laboratory and online	7.0	r = −0.01	.84[c]
Massé-Alarie et al[77] (2016)	22 (8)	n.a.	Chronic low back pain	n.a.	TSK-17	MET min/week (mean not specified)	GPAQ	n.a.	VAS	RCT	Clinical	7.8	r = 0.09	.69[c]
Miller et al[150] (2018)	52 (32)	67.4 (5.1)	Older adults	18.9	TSK-17	6743 steps/d	Hip accelerometer	1.4	QWBS-P	Cross-Sectional	Clinical	7.0	r = −0.54	<.001
Minetama et al[88] (2022)	71 (36)	71.6 (5.6)	Lumbar spinal stenosis	24.8	TSK-11	3601 steps/d	Pedometer	6.2	NRS	Cross-sectional	Clinical	7.0	r = −0.229	.055
Navarro-Ledesma et al[121] (2022)	41 (41)	52.6 (8.0)	Fibromyalgia	27.5 (6.9)	TSK-11	29.1 (18.2)	GLTEQ	n.a.		Cross-sectional	n.a.	7.0	r = −0.059	>.05
Norte et al[134] (2019)	77 (35)	21.6 (7.8)	Surgery (ACLR)	32.9 (6.0)	TSK-17	72.7 (34.9)	GLTEQ	91.4 (9.2)	KOOS-P	Cross-sectional	Laboratory	7.0	r = −0.312	<.05
Ohlman et al[151] (2018)	52 (33)	67.4 (5.1)	Older adults	18.8 (4.5)	TSK-11	n.a.	Hip accelerometer	n.a.		Cohort	Laboratory	8.0	ρ = −0.29	<.05
Olsson et al[135] (2014)	81 (12)	40.0 (9.6)	Achilles tendon rupture	35.9 (7.5)	TSK-17	2.9 (1.0)	SGPALS	n.a.		Cross-sectional	Clinical and laboratory	7.0	ρ = −0.275	.013
Ozer et al[146] (2022)	62 (30)	36.8 (6.1)	Asthma	39.6 (5.8)	TSK-17	2249 MET min/wk (1333)	IPAQ	n.a.		Cross-sectional	n.a.	8.0	r = −0.889	.001

Özlü and Akdeniz Leblebicier[141] (2022)	45 (45)	54.22 (8.2)	Rheumatoid arthritis	38.5 (15.7)	TSK-17	1980.7 MET min/wk (1104.8)	IPAQ	2.4 (1.7)	VAS	Controlled trial	Clinical	7.3	*r* = 0.152	.32
Palstam et al[67] (2014)	73 (73)	50.4 (9.3)	Fibromyalgia	9.7 (6.08)	FABQ	4.47 (3.64)	LTPAI	58.07 (20.29)	FIQ-pain	Cross-sectional	Clinical	7.0	*r* for MPA = 0.03	.80[c]
													r for VPA = −0.14	.23[c]
Pastor-Mira et al[128] (2020)	274 (274)	51.8 (9.1)	Fibromyalgia	27.5 (7.1)	TSK-11	3922.40 steps/wk	Pedometer	6.51 (1.62)	NRS	Cohort	Clinical	8.0	*r* = 0.01	>.05
Pazzinatto et al[89] (2022)	92 (92)	n.a. (18–35)	Patellofemoral pain	35.3 (6.8)	TSK-17	7.8 (1.5)	BHPAQ	5.1 (2.1)	VAS	Cross-sectional	Clinical	8.0	ρ = −0.14	.18[c]
Pedler et al[122] (2018)	103 (74)	39.7 (13.9)	Whiplash injury	26	TSK-11	9.9% of active time (8.2)	Trunk accelerometer	4.0 (2.4)	VAS	Cross-sectional	n.a.	7.0	*r* = 0.140	>.05
Peres et al[142] (2023)	50 (38)	62.8 (10.9)	Rheumatoid arthritis	42.7 (7.8)	TSK-17	3706.9 MET min/wk (2958.3)	SQUASH	n.a.		Cross-sectional	n.a.	8.0	*r* = −0.251	.055
	50 (31)	46.8 (12.3)	Spondyloarthritis	40.8 (7.6)	TSK-17	5614.9 MET min/wk (3681.7)	SQUASH						*r* = 0.170	.188
Polaski et al[78] (2021)	38 (26)	37.6 (13.4)	Chronic low back pain	19.5 (12.6)	FABQ	2821 MET min/wk;11,441 steps/d	IPAQ; accelerometer	3.1	VAS	RCT	Clinical	8.4	n.a.	n.a.
													r = −0.033	.42
Priore et al[73] (2020)	50 (37)	22.4 (3.9)	Patellofemoral pain	36.7	TSK-17	3088 MET min/wk	IPAQ	n.a.	VAS	RCT	Laboratory	9.2	*r* = −0.251[d]	.072[d]
Rabey et al[79] (2017)	266 (157)	51 (n.a.)	Chronic low back pain	14.3	FABQ	114 (0-302.5) MET min/wk	IPAQ	5.8	NRS	Cohort	Clinical	8.0	*r* = 0.077	.25[c]
Roaldsen et al[104] (2009)	98 (62)	76 (60–86)	Leg ulcer	12	FABQ	2.6	SGPALS	1.3	VRS	Cross-sectional	Online	8.0	*r* = −0.39[d]	7.1×10^{-5}[c]

Sandal et al[80] (2021)	461 (255)	45.7 (14.7)	Chronic low back pain	10.3 (5.4)	FABQ	n.a.	SGPALS	4.9 (1.9)	NRS	RCT	Online	8.8	*r* = −0.024	.66[c]
Saulicz et al[107] (2016)	105 (105)	n.a.	Older adults	45.2 (15.6)	KCS	n.a.	BHPAQ	n.a.		Cross-sectional	Online	7.0	*r* = −0.577[d]	<.001[d]
Sertel et al[123] (2021)	163 (76)	71.4 (6.0)	Chronic pain	44.4 (7.7)	TSK-17	171.3 (76.2)	PASE	n.a.	VAS	Cross-sectional	Home visit	7.0	*r* = −0.021	>.05
Smulligan et al[143] (2023)	23 (11)	14.9 (1.8)	Concussion (no persistent symptoms)	37	TSK-17	10,545 steps/d (3405)	Wrist accelerometer	n.a.		Cohort	Clinical	8.0	*r* for steps = −0.18	.41
						4.4 sessions/wk (1.9)	Wrist accelerometer						*r* for frequency = −0.34	.12
						46.8 min/session (26.2)	Wrist accelerometer						ρ for duration = 0.10	.67
	18 (9)	14.5 (2)	Concussion (persistent symptoms)	41	TSK-17	7047 steps/d (2499)	Wrist accelerometer	n.a.					*r* for steps = −0.60	.008
						2.3 sessions/wk (2.1)	Wrist accelerometer						*r* for frequency = −0.63	.05
						38 min/session (12)	Wrist accelerometer						ρ for duration = −0.12	.65
Spaderna et al[129] (2020)	61 (13)	67.5 (10.7)	Heart failure	1.5	FActS	2332 kcal/d	Hip accelerometer	n.a.		Cross-sectional	Laboratory	9.0	*r* = −0.28	<.05
Strandberg et al[74] (2022)	451[e] (n.a.)	n.a.	Cancer	n.a.	TSK-14	1.3 h of MVPA/wk (0.8)	Arm accelerometer	n.a.		RCT	Laboratory	8.8	*r* = −0.084[d]	.074[d]
Sütçü et al[144] (2021)	20 (10)	69.8 (9.4)	Parkinson disease	39.8 (7.4)	TSK-17	3078 steps/d	Arm accelerometer	n.a.		Cross-sectional	n.a.	6.0	*r* = −0.32[b]	.157

						2055 kcal/d (475)	Arm accelerometer						*r* = −0.54[b]	.013
Suttmiller et al[81] (2022)	126 (107)	32.69 (4.38)	Chronic pain	21.36 (5.53)	TSK-11	n.a.	JPAS	n.a.	Ad hoc questionnaire: “yes” and “no”	Cross-sectional	Online	8.0	*r* = 0.219	.014
Uritani et al[75] (2020)	167 (105)	62.2 (7.5)	Knee osteoarthritis	12.5	BFOMSO	7998 steps/d	Thigh accelerometer	5.7	NRS	Cross-sectional	n.a.	7.0	*r* = −0.163[d]	.035[d]
Verbunt et al[124] (2005)	123 (57)	44.1 (10.3)	Subacute low back pain	36.0	TSK-17	n.a.	Hip accelerometer	4.2	VAS	Cross-sectional	n.a.	7.0	ρ = 0.06	>.05
Wang et al[147] (2023)	223 (37)	72.35 (8.96)	Pulmonary condition (COPD)	20.72 (3.79)	BBQ	n.a.	IPAQ	n.a.		Cross-sectional	n.a.	7.0	*r* = −0.35	<.001
Wasiuk-Zowada et al[90] (2022)	80 (60)	45.5 (8.6)	Multiple sclerosis	36.6	TSK-17	5.1	BHPAQ	3.5	VAS	Cross-sectional	Clinical	7.0	*r* = −0.363	.001
Yuksel Karsli et al[91] (2021)	34 (12)	41 (n.a.)	Radiographic SpA	42	TSK-17	2203 min of LPA/d	Hip accelerometer	n.a.		Cross-sectional	Clinical	8.0	ρ = −0.16	.929
						210 min of MPA/d	Hip accelerometer						ρ = −0.158	.373
						0 min of VPA/d	Hip accelerometer						ρ = −0.394	.021
	33 (10)	33 (n.a.)	Nonradiographic SpA	36	TSK-17	2576 min of LPA/d	Hip accelerometer						ρ = −0.001	.997
						265 min of MPA/d	Hip accelerometer						ρ = 0.013	.947
						2 min of VPA/d	Hip accelerometer						ρ = −0.240	.209

Zadro et al[68] (2019)	60 (31)	68.3 (5.7)	Chronic low back pain	34.2 (5.9)	TSK-17	n.a.	RAPAQ	5 (1.7)	NRS	RCT	Laboratory	3.2	*r* for MPA = −0.18	.16[c]
													r for VPA = −0.12	.36[c]
Zelle et al[136] (2016)	487 (209)	51.6 (12.5)	Renal transplantation	n.a.	TSK-11	165 METs	MLTPAQ and TOAQ	n.a.		Cross-sectional	n.a.	7.0	*r* = −0.22	<.001

[a]Data for fear of movement, physical activity, and level of pain are scores unless otherwise indicated. ACLR = anterior cruciate ligament reconstruction; AHS = Australian Health Survey; BBQ = Breathlessness Beliefs Questionnaire (17–85), β = beta; BFOMSO = Brief Fear of Movement Scale for Osteoarthritis (6–24); BHPAQ = Baecke Habitual Physical Activity Questionnaire (3–15); BPI-S = Brief Pain Inventory–Severity (1–10); COPD = chronic obstructive pulmonary disease; CVD = cardiovascular disease; FABQ = Fear Avoidance Beliefs Questionnaire (0n–24); FActS = Fear of Activity in Situations (0–30); FIQ-pain = Fibromyalgia Impact Questionnaire–Pain; FQPA = Freiburger Questionnaire on Physical Activity; GLTEQ = Godin Leisure Time Exercise Questionnaire (0–119); GPAQ = Global Physical Activity Questionnaire; GRS = graphic rating scale (0–10); IPAQ = short form of the International Physical Activity Questionnaire; JPAS = Jurka Physical Activity Scale; KCS = Kinesiophobia Causes Scale (0–100); KOOS-P = Knee Injury and Osteoarthritis Outcome Score–Pain (0–100); LPA = light physical activity; LTPAI = Leisure Time Physical Activity Index; LTPAQ = Leisure Time Physical Activity Questionnaire; MET = metabolic equivalent task; n.a. = not available; MLTPAQ = Minnesota Leisure Time Physical Activity Questionnaire; MPA = moderate physical activity; MSK = musculoskeletal; MVPA = moderate to vigorous physical activity; NRS = numeric rating scale (0–10); OKS = Oxford Knee Score–Pain (0–28); PAQE = Physical Activity Questionnaire for the Elderly (0–3); PASE = Physical Activity Scale for the Elderly; QWBS-P = Quality of Well-Being Scale–Pain (0–5); *r* = Pearson correlation coefficient; RAPAQ = Rapid Assessment of Physical Activity Questionnaire; RCT = randomized controlled trial; ρ = Spearman correlation coefficient; SF-36 = 36-Item Short Form Health Survey (0–100); SGPALS = Saltin-Grimby Physical Activity Level Scale (1–4); SpA = axial spondyloarthritis; SQUASH = Short Questionnaire to Assess Health Enhancing Physical Activity; TAS = Tegner Activity Scale (0–10); TOAQ = Tecumseh Occupational Activity Questionnaire; TSK = Tampa Scale for Kinesiophobia (adaptation of the TSK for patients with coronary artery disease [TSK-Heart], scored from 17–68; 7-item TSK [TSK-7], scored from 7–28; 11-item TSK [TSK-11], scored from 11–44; 13-item TSK [TSK-13], scored from 13–52; 14-item TSK [TSK-14], scored from 14–56; 17-item TSK [TSK-17], scored from 17–68; UCLA = University of California–Los Angeles activity score (1–10); VAS = visual analog scale (0–10); VPA = vigorous physical activity; VRS = verbal rating scale for pain assessment (0–5).
[b]When the Pearson correlation coefficient (*r*) was not reported in an article, but the exact *P* value and sample size were available and it was possible to know the sign of the correlation based on the information provided in the article, the *r* value was computed using an ad hoc R code (Suppl. Code 1A).
[c]When exact *P* values were not reported in an article but the sample size and Pearson correlation coefficient were available, the exact *P* value was computed using an ad hoc R code (Suppl. Code 1B).
[d]Obtained by email from the authors.
[e]Number of participants used to calculate the correlation, according to the email sent by the authors.

Supplementary Figure 1. Heterogeneity variance.

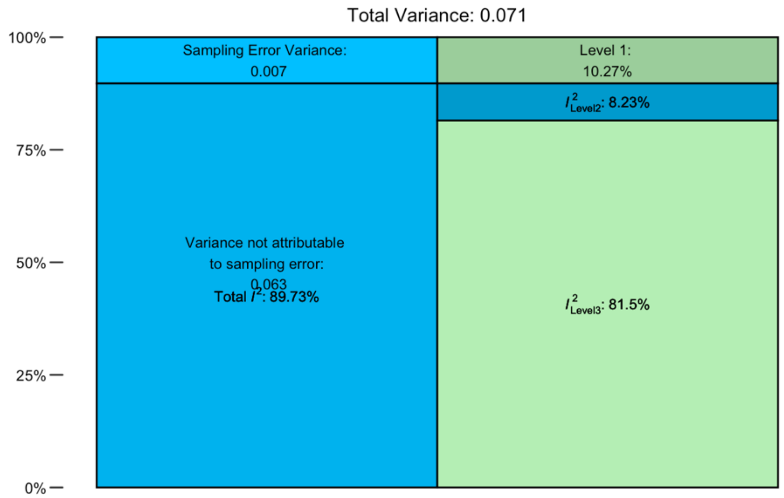

Supplementary Figure 2. Secondary meta-analysis based on Pearson's rho estimates.

Study	Cluster	Total	Weight	Correlation IV, Random, 95% CI
Atici	sample_1	254	9.0%	-0.34 [-0.45; -0.23]
Barchek, 2021	sample_2	19	3.6%	0.12 [-0.35; 0.54]
Barchek, 2021	sample_2	19	3.6%	-0.13 [-0.55; 0.34]
Carvalho, 2017	sample_3	119	4.5%	-0.15 [-0.32; 0.03]
Carvalho, 2017	sample_3	119	4.5%	-0.13 [-0.30; 0.05]
Gonzalez de La Flor, 2022	sample_4	42	7.5%	0.20 [-0.11; 0.48]
Knapik, 2019	sample_5	135	8.7%	-0.80 [-0.85; -0.73]
Leonhardt, 2009	sample_6	449	5.3%	-0.09 [-0.18; -0.00]
Leonhardt, 2009	sample_6	338	4.0%	-0.10 [-0.20; 0.01]
Ohlman, 2018	sample_7	52	7.8%	-0.29 [-0.52; -0.02]
Olsson, 2014	sample_8	81	8.3%	-0.27 [-0.46; -0.05]
Pazzinato, 2022	sample_9	92	8.4%	-0.14 [-0.34; 0.07]
Smulligan, 2023	sample_10	23	4.2%	0.10 [-0.33; 0.49]
Smulligan, 2023	sample_10	18	3.1%	-0.12 [-0.56; 0.37]
Verbunt, 2005	sample_11	123	8.7%	0.06 [-0.12; 0.23]
Yuksel Karsli, 2019	sample_12	34	1.5%	-0.39 [-0.64; -0.06]
Yuksel Karsli, 2019	sample_12	34	1.5%	-0.16 [-0.47; 0.19]
Yuksel Karsli, 2019	sample_12	34	1.5%	-0.16 [-0.47; 0.19]
Yuksel Karsli, 2019	sample_12	33	1.5%	-0.01 [-0.35; 0.33]
Yuksel Karsli, 2019	sample_12	33	1.5%	0.01 [-0.33; 0.35]
Yuksel Karsli, 2019	sample_12	33	1.5%	-0.24 [-0.54; 0.11]
**Total (95% CI)**		**2084**	**100.0%**	**-0.20 [-0.38; -0.00]**
**Prediction interval**				**[-0.71; 0.45]**

Heterogeneity: $Tau^2 = 0.0982$; $Chi^2 = 146.02$, df = 20 ($P < 0.01$); $I^2 = 86\%$

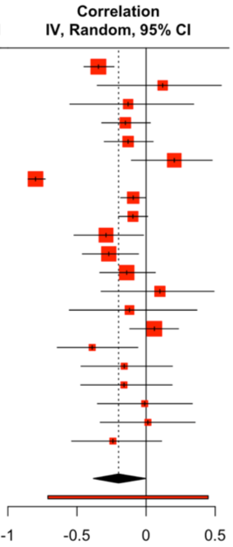

Supplementary Figure 3. Subgroup meta-analysis: Differences by physical activity (PA) measurement instrument.

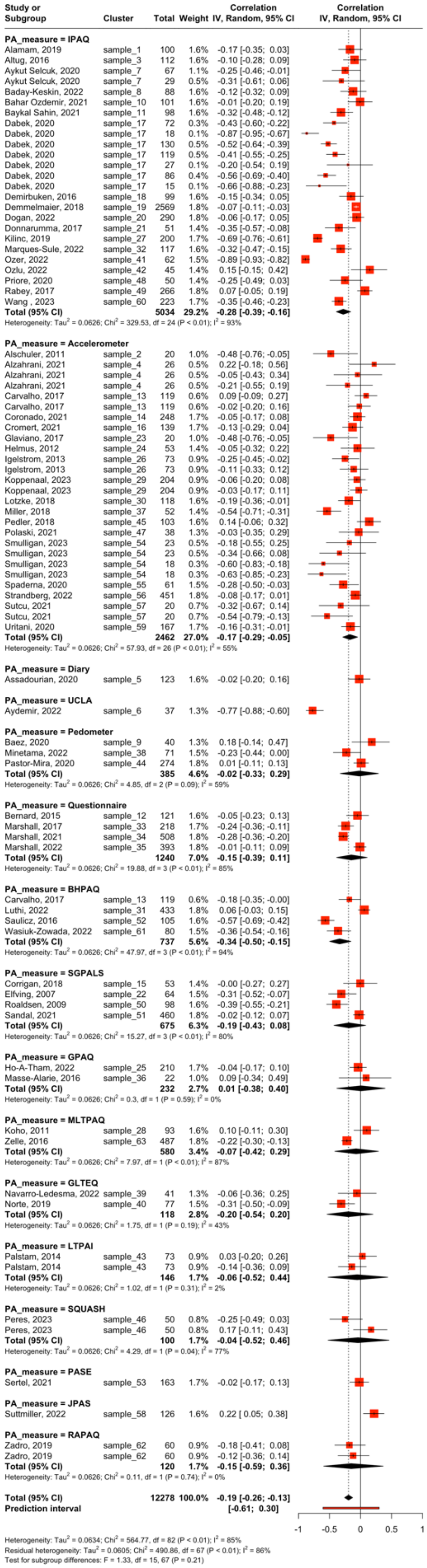

Supplementary Figure 4. Subgroup meta-analysis: Differences by physical activity (PA) outcome

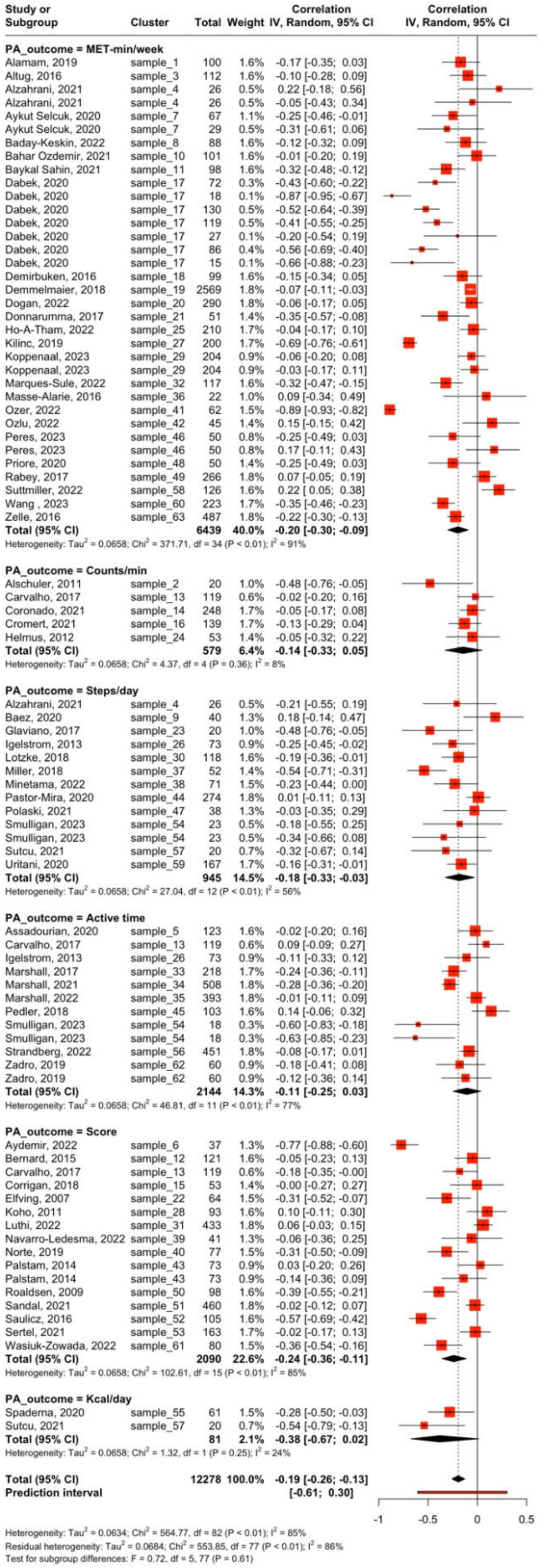

Supplementary Figure 5. Subgroup meta-analysis: Differences by fear of movement (FoM) measurement instrument.

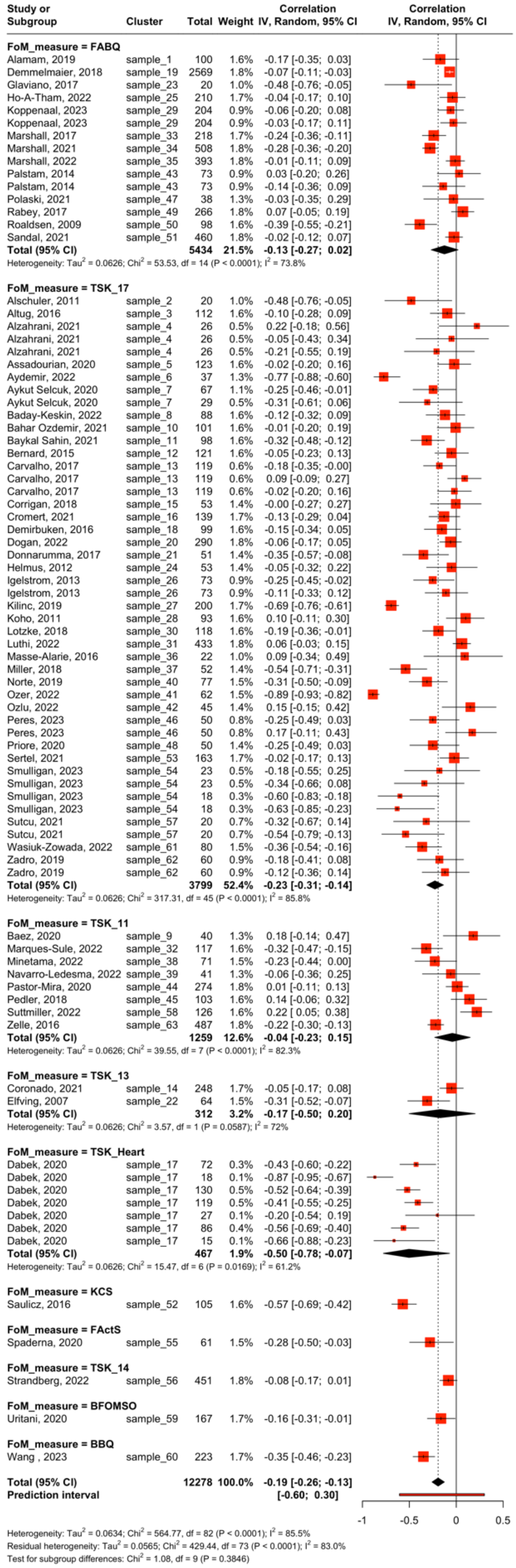

Supplementary Figure 6. Meta-regressions testing the influence of age (A; 72 studies), the proportion of women (B; 72 studies), and pain intensity (C; 49 studies) on the relationship between fear of movement and physical activity.

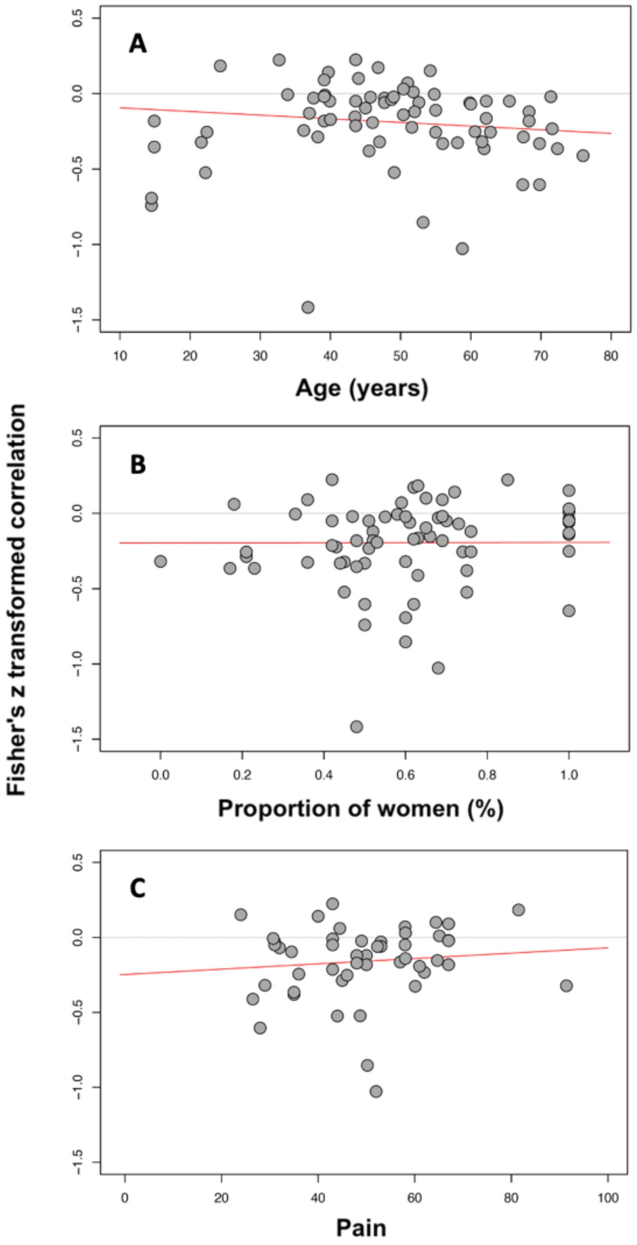

References

29. Bahar Özdemir Y. Investigation of low back pain in the white-collar population working from home due to the COVID-19 pandemic. *J Phys Med Rehabil Sci.* 2021;24(2):135-142. https://doi.org/10.31609/jpmrs.2021-81527

62. Assadourian M, Bailly F, Letellier P, et al. Criteria for inclusion in programs of functional restoration for chronic low back pain: pragmatic study. *Ann Phys Rehabil Med.* 2020;63(3):189-194. https://doi.org/10.1016/j.rehab.2019.06.019

63. Baez SE, Hoch MC, Hoch JM. Psychological factors are associated with return to pre-injury levels of sport and physical activity after ACL reconstruction. *Knee Surg Sports Traumatol Arthrosc.* 2020;28(2):495-501. https://doi.org/10.1007/s00167-019-05696-9

64. Luthi F, Vuistiner P, Favre C, Hilfiker R, Léger B. Avoidance, pacing, or persistence in multidisciplinary functional rehabilitation for chronic musculoskeletal pain: an observational study with cross-sectional and longitudinal analyses. *PLOS One.* 2018;13(9):e0203329. https://doi.org/10.1371/journal.pone.0203329

65. Alzahrani H, Mackey M, Stamatakis E, Shirley D. Wearables-based walking program in addition to usual physiotherapy care for the management of patients with low back pain at medium or high risk of chronicity: a pilot randomized controlled trial. *PLOS One.* 2021;16(8):e0256459. https://doi.org/10.1371/journal.pone.0256459

66. Koppenaal T, van Dongen JM, Kloek CJ, et al. Effectiveness and cost-effectiveness of a stratified blended physiotherapy intervention compared with face-to-face physiotherapy in patients with nonspecific low back pain: cluster randomized controlled trial. *J Med Internet Res*. 2023;25:e43034. https://doi.org/10.2196/43034

67. Palstam A, Larsson A, Bjersing J, et al. Perceived exertion at work in women with fibromyalgia: explanatory factors and comparison with healthy women. *J Rehabil Med.* 2014;46(8):773-780. https://doi.org/10.2340/16501977-1843

68. Zadro JR, Shirley D, Simic M, et al. Video-game-based exercises for older people with chronic low back pain: a randomized controlled trial (GAMEBACK). *Phys Ther.* 2019;99(1):14-27. https://doi.org/10.1093/ptj/pzy112

69. Marshall PW, Schabrun S, Knox MF. Physical activity and the mediating effect of fear, depression, anxiety, and catastrophizing on pain related disability in people with chronic low back pain. *PLOS One*. 2017;12(7). https://doi.org/10.1371/journal.pone.0180788

70. Marshall PW, Morrison, NM V, Mifsud A, Gibbs M, Khan N, Meade T. The moderating effect of treatment engagement on fear-avoidance beliefs in people with chronic low back pain. *Clin J Pain*. 2021;37(12):872-880. https://doi.org/10.1097/AJP.0000000000000991

71. Marshall PW, Morrison NMV, Gibbs M, Schabrun SM. The effect of exercise engagement on low back disability at 12-months is mediated by pain and catastrophizing in a community sample of people with chronic low back pain. *Behav Res Ther*. 2022;159:104205. https://doi.org/10.1016/j.brat.2022.104205

72. Bernard P, Ninot G, Bernard PL, et al. Effects of a six-month walking intervention on depression in inactive post-menopausal women: a randomized controlled trial. *Aging Ment Health*. 2015;19(6):485-492. https://doi.org/10.1080/13607863.2014.948806

73. Priore LB, Lack S, Garcia C, Azevedo FM, de Oliveira Silva D. Two weeks of wearing a knee brace compared with minimal intervention on kinesiophobia at 2 and 6 weeks in people with patellofemoral pain: a randomized controlled trial. *Arch Phys Med Rehabil*. 2020;101(4):613-623. https://doi.org/10.1016/j.apmr.2019.10.190

74. Strandberg E, Bean C, Vassbakk-Svindland K, et al. Who makes it all the way? Participants vs. decliners, and completers vs. drop-outs, in a 6-month exercise trial during cancer treatment. Results from the Phys-Can RCT. *Support Care Cancer*. 2022;30(2):1739-1748. https://doi.org/10.1007/s00520-021-06576-0

75. Uritani D, Kasza J, Campbell PK, Metcalf B, Egerton T. The association between psychological characteristics and physical activity levels in people with knee osteoarthritis: a cross-sectional analysis. *BMC Musculoskelet Disord*. 2020;21(1):269. https://doi.org/10.1186/s12891-020-03305-2

76. Alamam DM, Moloney N, Leaver A, Alsobayel HI, Mackey MG. Multidimensional prognostic factors for chronic low back pain-related disability: a longitudinal study in a Saudi population. *Spine J*. 2019;19(9),1548-1558. https://doi.org/10.1016/j.spinee.2019.05.010

77. Massé-Alarie H, Beaulieu LD, Preuss R, Schneider C. Influence of paravertebral muscles training on brain plasticity and postural control in chronic low back pain. *Scand J Pain*. 2016;12(1):74-83. https://doi.org/10.1016/j.sjpain.2016.03.005

78. Polaski AM, Phelps AL, Smith TJ, et al. Integrated meditation and exercise therapy: a randomized controlled pilot of a combined nonpharmacological intervention focused on reducing disability and pain in patients with chronic low back pain. *Pain Med*. 2021;22(2):444-458. https://doi.org/10.1093/pm/pnaa403

79. Rabey M, Smith A, Beales D, Slater H, O'Sullivan P. Multidimensional prognostic modelling in people with chronic axial low back pain. *Clin J Pain*. 2017;33(10):877-891. https://doi.org/10.1097/AJP.0000000000000478

80. Sandal LF, Bach K, Øverås CK, et al. Effectiveness of app-delivered, tailored self-management support for adults with lower back pain–related disability: a SELFBACK randomized clinical trial. *JAMA Intern Med*. 2021;181(10):1288-1296. https://doi:10.1001/jamainternmed.2021.4097

81. Suttmiller AMB, Cavallario JM, Baez SE, Martinez JC, McCann RS. Perceived instability, pain, and psychological factors for prediction of function and disability in individuals with chronic ankle instability. *J Athl Train.* 2022;57(11-12):1048-1054. https://doi.org/10.4085/1062-6050-0605.21

82. Demmelmaier I, Björk A, Dufour AB, Nordgren B, Opava, CH. Trajectories of fear-avoidance beliefs on physical activity over two years in people with rheumatoid arthritis. *Arthritis Care Res*. 2018;70(5):695-702. http://doi.org/10.1002/acr.23419

83. Ho-A-Tham N, Struyf N, Ting-A-Kee B, de Almeida Mello J, Vanlandewijck Y, Dankaerts W. Physical activity, fear avoidance beliefs and level of disability in a multi-ethnic female population with chronic low back pain in Suriname: a population-based study. *PLOS One*. 2022;17(10):e0276974. https://doi.org/10.1371/journal.pone.0276974

84. Aydemir B, Huang CH, Foucher KC. Strength and physical activity in osteoarthritis: the mediating role of kinesiophobia. *J Orthop Res.* 2022;40(5):1135-1142. http://doi.org/10.1002/jor.25151

85. Baday-Keskin D, Ekinci B. The relationship between kinesiophobia and health-related quality of life in patients with rheumatoid arthritis: a controlled cross-sectional study. *Joint Bone Spine.* 2022;89(2):105275. http://doi.org/10.1016/j.jbspin.2021.105275

86. Dąbek J, Knapik A, Gallert-Kopyto W, Brzęk AM, Piotrkowicz J, Gąsior Z. Fear of movement (kinesiophobia) - an underestimated problem in Polish patients at various stages of coronary artery disease. *Ann Agric Environ Med.* 2020;27(1):56-60. http://doi.org/10.26444/aaem/106143

87. Knapik A, Dąbek J, Brzęk A. Kinesiophobia as a problem in adherence to physical activity recommendations in elderly Polish patients with coronary artery disease. *Patient Prefer Adherence.* 2019;13:2129-2135. http://doi.org/10.2147/PPA.S216196

88. Minetama M, Kawakami M, Teraguchi M, et al. Associations between psychological factors and daily step count in patients with lumbar spinal stenosis. *Physiother Theory Pract.* 2022;38(10):1519-1527. http://doi.org/10.1080/09593985.2020.1855685

89. Pazzinatto MF, Silva DO, Willy RW, Azevedo FM, Barton CJ. Fear of movement and (re)injury is associated with condition specific outcomes and health-related quality of life in women with patellofemoral pain. *Physiother Theory Pract.* 2022;38(9):1254-1263. http://doi.org/10.1080/09593985.2020.1830323

90. Wasiuk-Zowada D, Brzęk A, Krzystanek E, Knapik A. Kinesiophobia in people with multiple sclerosis and its relationship with physical activity, pain and acceptance of disease. *Medicina.* 2022;58(3):414. http://doi.org/10.3390/medicina58030414

91. Yuksel Karsli T, Bayraktar D, Ozer Kaya D, et al. Comparison of physical activity levels among different sub-types of axial spondyloarthritis patients and healthy controls. *Mod Rheumatol.* 2021;31(6):1202-1207. http://doi.org/10.1080/14397595.2021.1891676

104. Roaldsen KS, Elfving B, Stanghelle JK, Talme T, Mattsson E. Fear-avoidance beliefs and pain as predictors for low physical activity in patients with leg ulcer. *Physiother Res Int*. 2009;14(3):167-180. https://doi.org/10.1002/pri.433

107. Saulicz M, Saulicz E, Knapik A, et al. Impact of physical activity and fitness on the level of kinesiophobia in women of perimenopausal age. *Prz Menopauzalny*. 2016;15(2):104-111. https://doi.org/10.5114/pm.2016.61193

111. Alschuler KN, Hoodin F, Murphy SL, Rice J, Geisser ME. Factors contributing to physical activity in a chronic low back pain clinical sample: a comprehensive analysis using continuous ambulatory monitoring. *Pain*. 2011;152(11):2521-2527. https://doi.org/10.1016/j.pain.2011.07.017

112. Altuğ F, Ünal A, Kilavuz G, Kavlak E, Çitişli V, Cavlak U. Investigation of the relationship between kinesiophobia, physical activity level and quality of life in patients with chronic low back pain. *J Back Musculoskelet Rehabil.* 2016;29(3):527-531. https://doi.org/10.3233/BMR-150653

113. Carvalho FA, Maher CG, Franco MR, et al. Fear of movement is not associated with objective and subjective physical activity levels in chronic nonspecific low back pain. *Arch Phys Med Rehabil.* 2017;98(1):96-104. http://doi.org/10.1016/j.apmr.2016.09.115

114. Demirbüken İ, Özgül B, Kuru Çolak T, Aydoğdu O, Sarı Z, Yurdalan SU. Kinesiophobia in relation to physical activity in chronic neck pain. *J Back Musculoskelet Rehabil.* 2016;29(1):41-47. http://doi.org/10.3233/BMR-150594

115. Elfving B, Andersson T, Grooten WJ. Low levels of physical activity in back pain patients are associated with high levels of fear-avoidance beliefs and pain catastrophizing. *Physiother Res Int.* 2007;12(1):14-24. http://doi.org/10.1002/pri.355

116. González de la Flor Á, García Pérez de Sevilla G, Domíngez Balmaseda D, Martín Vera D, Montero Martínez M, Del Blanco Muñiz JÁ. Relationship between self-efficacy and headache impact, anxiety, and physical activity levels in patients with chronic tension-type headache: an observational study. *Behav Neurol.* 2022;2022:8387249. http://doi.org/10.1155/2022/8387249

117. Helmus M, Schiphorst Preuper HR, Hof AL, Geertzen JH, Reneman MF. Psychological factors unrelated to activity level in patients with chronic musculoskeletal pain. *Eur J Pain.* 2012;16(8):1158-1165. http://doi.org/10.1002/j.1532-2149.2011.00109.x

118. Huijnen IP, Verbunt JA, Peters ML, Seelen HA. Is physical functioning influenced by activity-related pain prediction and fear of movement in patients with subacute low back pain? *Eur J Pain.* 2010;14(6):661-666. http://doi.org/10.1016/j.ejpain.2009.10.014

119. Koho P, Orenius T, Kautiainen H, Haanpää M, Pohjolainen T, Hurri H. Association of fear of movement and leisure-time physical activity among patients with chronic pain. *J Rehabil Med.* 2011;43(9):794-799. http://doi.org/10.2340/16501977-0850

120. Lotzke H, Jakobsson M, Gutke A, et al. Patients with severe low back pain exhibit a low level of physical activity before lumbar fusion surgery: a cross-sectional study. *BMC Musculoskelet Disord.* 2018;19(1):365. http://doi.org/10.1186/s12891-018-2274-5

121. Navarro-Ledesma S, Pruimboom L, Lluch E, Dueñas L, Mena-Del Horno S, Gonzalez-Muñoz A. The relationship between daily physical activity, psychological factors, and vegetative symptoms in women with fibromyalgia: a cross-sectional observational study. *Int J Environ Res Public Health.* 2022;19(18):11610. http://doi.org/10.3390/ijerph191811610

122. Pedler A, Kamper SJ, Maujean A, Sterling M. Investigating the fear avoidance model in people with whiplash: the association between fear of movement and in vivo activity. *Clin J Pain.* 2018;34(2):130-137. http://doi.org/10.1097/AJP.0000000000000524

123. Sertel M, Aydoğan Arslan S, Tütün Yümin E, Demirci CS, Tarsuslu Şimşek T. Investigation of the relationship between physical activity, kinesiophobia and fear of falling in older adults with chronic pain. *Somatosens Mot Res.* 2021;38(3):241-247. http://doi.org/10.1080/08990220.2021.1958774

124. Verbunt JA, Sieben JM, Seelen HA, et al. Decline in physical activity, disability and pain-related fear in sub-acute low back pain. *Eur J Pain.* 2005;9(4):417-425. http://doi.org/10.1016/j.ejpain.2004.09.011

125. Corrigan P, Cortes DH, Pontiggia L, Silbernagel KG. The degree of tendonitis is related to symptom severity and physical activity levels in patients with midportion Achilles tendinopathy. *Int J Sports Phys Ther.* 2018;13(2):196-207. http://doi.org/10.26603/ijspt20180196

126. Glaviano NR, Baellow A, Saliba S. Physical activity levels in individuals with and without patellofemoral pain. *Phys Ther Sport.* 2017;27:12-16. https://doi.org/10.1016/j.ptsp.2017.07.002

127. Leonhardt C, Lehr D, Chenot JF, et al. Are fear-avoidance beliefs in low back pain patients a risk factor for low physical activity or vice versa? A cross-lagged panel analysis. *Psychosoc Med.* 2009;6:Doc01. https://doi.org/10.3205/psm000057

128. Pastor-Mira, MA, López-Roig S, Peñacoba C, Sanz-Baños Y, Lledó A, Velasco L. Predicting walking as exercise in women with fibromyalgia from the perspective of the theory of planned behavior. *Women Health*. 2020;60(4),412-425. https://doi.org/10.1080/03630242.2019.1662869

129. Spaderna H, Hoffman JM, Hellwig S, Brandenburg VM. Fear of physical activity, anxiety, and depression: barriers to physical activity in outpatients with heart failure? *Eur J Health Psychol.* 2020;27(1):3-13. http://doi.org/10.1027/2512-8442/a000042

130. Baykal Şahin H, Kalaycıoğlu E, Şahin M. The effect of cardiac rehabilitation on kinesiophobia in patients with coronary artery disease. *Turk J Phys Med Rehabil.* 2021;67(2):203-210. http://doi.org/10.5606/TFTRD.2021.5164

131. Marques-Sule E, Söderlund A, Almenar L, Espí-López GV, López-Vilella R, Bäck M. Influence on kinesiophobia by disability, physical, and behavioural variables after a heart transplantation. *Eur J Cardiovasc Nurs.* 2022;21(6):537-543. http://doi.org/10.1093/eurjcn/zvab134

132. Barchek AR, Dlugonski D, Baez SE, Hoch MC, Hoch J. The relationship between injury-related fear and physical activity in people with a history of anterior cruciate ligament reconstruction. *Phys Ther Sport*. 2021;50:201-205. https://doi.org/10.1016/j.ptsp.2021.05.010

133. Donnarumma P, Presaghi F, Tarantino R, Fragale M, Rullo M, Delfini R. The impact of pelvic balance, physical activity, and fear-avoidance on the outcome after decompression and instrumented fusion for degenerative lumbar stenosis. *Eur Spine J.* 2017;26(2):428-433. http://doi.org/10.1007/s00586-016-4644-8

134. Norte GE, Solaas H, Saliba SA, Goetschius J, Slater LV, Hart JM. The relationships between kinesiophobia and clinical outcomes after ACL reconstruction differ by self-reported physical activity engagement. *Phys Ther Sport.* 2019;40:1-9. http://doi.org/10.1016/j.ptsp.2019.08.002

135. Olsson N, Karlsson J, Eriksson BI, Brorsson A, Lundberg M, Silbernagel KG. Ability to perform a single heel-rise is significantly related to patient-reported outcome after Achilles tendon rupture. *Scand J Med Sci Sports.* 2014;24(1):152-158. http://doi.org/10.1111/j.1600-0838.2012.01497.x

136. Zelle DM, Corpeleijn E, Klaassen G, Schutte E, Navis G, Bakker SJL. Fear of movement and low self-efficacy are important barriers in physical activity after renal transplantation. *PLOS One.* 2016;11(2):e0147609. http://doi.org/10.1371/journal.pone.0147609

137. Coronado RA, Robinette PE, Henry AL, et al. Bouncing back after lumbar spine surgery: early postoperative resilience is associated with 12-month physical function, pain interference, social participation, and disability. *Spine J.* 2021;21(1):55-63. http://doi.org/10.1016/j.spinee.2020.07.013

138. Aykut Selçuk M, Karakoyun A. Is there a relationship between kinesiophobia and physical activity level in patients with knee osteoarthritis? *Pain Med.* 2020;21(12):3458-3469. http://doi.org/10.1093/pm/pnaa180

139. Doğan N, Taşci S. Pain, physical activity, and kinesiophobia levels in individuals with knee osteoarthritis: a cross-sectional study. *Turk Klin J Nurs Sci*. 2022;14(4):1144-1154. https://doi:10.5336/nurses.2022-89904

140. Kilinç H, Karahan S, Atilla B, Kinikli Gİ. Can fear of movement, depression and functional performance be a predictor of physical activity level in patients with knee osteoarthritis? *Arch Rheumatol.* 2019;34(3):274-280. http://doi.org/10.5606/ArchRheumatol.2019.7160

141. Özlü A, Akdeniz Leblebicier M. Does remission in rheumatoid arthritis bring kinesiophobia, quality of life, fatigue, and physical activity closer to normal? *Arch Rheumatol*. 2022;37(4):603-612. http://doi.org/10.46497/ArchRheumatol.2022.9552

142. Peres D, Tordi N, Demartino AM, Cheng JL, Sagawa Jr Y, Prati C. Relationships between physical activity levels and disease activity, functional disability and kinesiophobia in chronic rheumatic diseases. *Sci Sports*. 2023;38(5-6):607-615. https://doi.org/10.1016/j.scispo.2022.07.013

143. Smulligan KL, Wingerson MJ, Seehusen CN, Little CC, Wilson JC, Howell DR. More physical activity is correlated with reduction in kinesiophobia for adolescents with persistent symptoms after concussion. *J Sport Rehabil*. 2023;32(2):196-202. https://doi.org/10.1123/jsr.2022-0193

144. Sütçü G, Ayvat E, Kilinç M. Effects of fatigue and kinesiophobia on functional capacity, physical activity and quality of life in Parkinson's disease. *Int J Rehabil Res.* 2021;44(1):65-68. http://doi.org/10.1097/MRR.0000000000000449

145. Igelström H, Emtner M, Lindberg E, Åsenlöf P. Physical activity and sedentary time in persons with obstructive sleep apnea and overweight enrolled in a randomized controlled trial for enhanced physical activity and healthy eating. *Sleep Breath*. 2013;17(4):1257-1266. https://doi.org/10.1007/s11325-013-0831-6

146. Ozer AY, Karaca S, Senocak E, Oguz S, Polat MG. Does kinesiophobia limit physical activity and quality of life in asthmatic patients? *Int J Rehabil Res.* 2022;45(3):230-236. http://doi.org/10.1097/MRR.0000000000000534

147. Wang J, Bai C, Zhang Z, Chen O. The relationship between dyspnea-related kinesiophobia and physical activity in people with COPD: cross-sectional survey and mediated moderation analysis. *Heart Lung*. 2023;59:95-101. https://doi.org/10.1016/j.hrtlng.2023.02.007

148. Crommert ME, Flink I, Gustavsson C. Predictors of disability attributed to symptoms of increased interrecti distance in women after childbirth: an observational study. *Phys Ther*. 2021;101(6):pzab064. https://doi.org/10.1093/ptj/pzab064

149. Atıcı E, Girgin N, Çevik Saldıran T. The effects of social isolation due to COVID-19 on the fear of movement, falling, and physical activity in older people. *Australas J Ageing.* 2022;41(3):407-413. http://doi.org/10.1111/ajag.1306390

150. Miller L, Ohlman T, Naugle KM. Sensitivity to physical activity predicts daily activity among pain-free older adults. *Pain Med.* 2018;19(8):1683-1692. http://doi.org/10.1093/pm/pnx251

151. Norte GE, Solaas H, Saliba SA, Goetschius J, Slater LV, Hart JM. The relationships between kinesiophobia and clinical outcomes after ACL reconstruction differ by self-reported physical activity engagement. *Phys Ther Sport.* 2019;40:1-9. http://doi.org/10.1016/j.ptsp.2019.08.002
